# Covalent immobilization of anthocyanins onto cellulose nanofibrils for intelligent food packaging films

**DOI:** 10.1016/j.fochx.2026.104175

**Published:** 2026-07-06

**Authors:** Jiawei Zheng, Yang Zhang, Xidong Li, Qinghua Xu, Liqiang Jin

**Affiliations:** State Key Laboratory of Green Papermaking and Resource Recycling, Qilu University of Technology (Shandong Academy of Sciences), Jinan 250353, China

**Keywords:** Anthocyanins, Cellulose nanofibrils, Gelatin, Color indicator films

## Abstract

To address the instability and moisture-induced leaching of anthocyanins (ACNs), an intelligent packaging film was fabricated by covalently grafting ACNs onto dialdehyde cellulose nanofibrils (DACNFs) to produce a stable anthocyanin–DACNF (ADNF) nanocomposite, which was subsequently incorporated into a gelatin (GE) matrix. Compared with the GE film, the GE-ADNF films exhibited superior tensile strength (up to 107.84 MPa), improved water resistance (water contact angle of ∼110°), UV-shielding, antioxidant activity (DPPH scavenging >70% after 12 h). Color stability was enhanced (ΔE <5 at 60 °C for 24 h). When monitoring pork samples at 25 °C, the film color change strongly correlated with total volatile basic nitrogen (TVB-N) and pH, effectively discriminating between fresh, sub-fresh, and spoiled states. The GE-ADNF film with 15% ADNF content show the highest sensitivity (R^2^ = 0.99 with TVB-N). This covalent immobilization strategy substantially improves stability of ACNs and GE-ADNF films, demonstrating high potential for real-time pork freshness monitoring.

## Highlights

• Covalently grafted ACNs onto DACNFs to form stable ADNF nanocomposites.
• GE-ADNF films showed high tensile strength, reaching up to 107.84 MPa.
• Improved water resistance with lower swelling, solubility, and higher WCA.
• Strong UV-shielding and sustained antioxidant activity in composite films.
• Real-time pork freshness monitoring correlated well with TVB-N values.

## Graphical abstract

**Figure:**
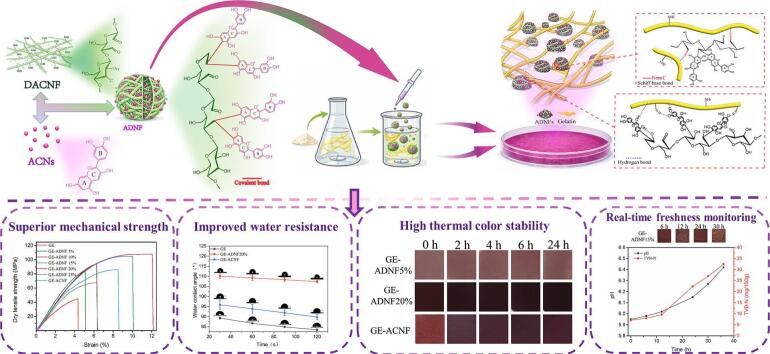

## 1 Introduction

Although traditional packaging methods are essential for physical protection, they show clear limitations in information delivery and consumer interaction (Yao et al., 2024). Therefore, intelligent packaging has become a major research focus (Wang et al., 2023). It combines sensing technologies and functional materials to detect changes in product parameters such as—such as pH shifts caused by microbial metabolism, temperature fluctuations that accelerate spoilage, accumulation of volatile gases (e.g., ammonia, biogenic amines, CO₂), and bacterial growth—thereby providing consumers with more complete and accurate food information (Zhang et al., 2025). Because of its intuitive, rapid, and convenient features, colorimetric pH-indicating packaging is widely applied in smart food packaging (En et al., 2025; Swarup & Jong-Whan, 2020), offering manufacturers and consumers real-time quality information of packaged food throughout storage and transportation. Safe and non-toxic natural colorants, such as alizarin, betalains, curcumin, shikonin, anthocyanins (ACNs) can be incorporated into biopolymer films to construct intelligent packaging for monitoring food freshness (Roy & Rhim, 2020). In particular, ACNs, a class of flavonoids, with notable antioxidant and antimicrobial activities (Guo, Li, et al., 2024), exhibit a broad pH-responsive discoloration behavior, which renders them suitable as colorimetric indicators (Mu et al., 2025). Therefore, incorporation of ACNs into biopolymer matrices has recently attracted increasing attention for developing active and intelligent packaging films or pH-responsive labels. Diverse biopolymers, including chitosan, cellulose, starch, gelatin (GE), and soy protein, can form ACN-loaded packaging films or labels through hydrogen bonding or electrostatic interactions, enabling real-time assessment of the freshness of packaged food (Karnwal et al., 2025).

However, despite their potential as natural indicators, the practical application of anthocyanins in intelligent packaging is limited because they readily undergo discoloration or degradation under environmental factors such as temperature, light, pH, and oxygen concentration (Zhang, Zou, et al., 2022). Moreover, indicator films based on natural polymers often exhibit high water solubility and hygroscopicity, limiting their use in high-humidity environments (Xu et al., 2024). Under such conditions, typically encountered in fresh meat or seafood packaging with high moisture content, hydrophilic ACNs tend to leach from the film matrix. This leaching not only reduces the colorimetric response but also raises potential food safety and flavor concerns (Zhang et al., 2023). In addition, it can weaken the film's visible color variation and decrease its sensitivity for monitoring food freshness, hindering real-time, reliable spoilage detection. Multiple strategies including microencapsulation and nanotechnology, such as emulsions (Alizadeh-Sani et al., 2020), nanocomposites (Ge et al., 2019; Liu et al., 2022), and liposomes (Zhang, Zhang, et al., 2022; Zhang, Zou, et al., 2022), have been explored to improve the stability of ACNs. Nevertheless, ACNs in these systems typically interact with stabilizers through non-covalent intermolecular forces, such as hydrogen bonding and electrostatic interactions (Xu et al., 2024)*.* These forces are often too weak to prevent leaching under high- humidity conditions, which in turn compromises color visualization.

Covalent immobilization offers a more robust alternative. This approach effectively enhances the stability of hydrophobic flavonoids upon grafting to polysaccharides and proteins (Sun et al., 2025; Yong & Liu, 2024; Yun et al., 2026). Catechins, including epicatechin, epicatechin gallate, epigallocatechin and epigallocatechin gallate have been grafted onto dialdehyde starch (DAS) to obtain DAS–catechin conjugates with enhanced thermal stability (Yong et al., 2022). Owing to its high specific surface area, Young's modulus, abundant hydroxyl groups, and excellent biocompatibility (Silva et al., 2020), nanocellulose is an ideal material for food packaging and a suitable substrate for covalent modification. It has been widely used as a substrate to prepare ACN-based intelligent films (Li et al., 2023; Wang et al., 2025). However, to the best of our knowledge, this is the first report on covalent immobilization of ACNs onto nanocellulose for intelligent food packaging applications.

To overcome the instability of ACNs in smart packaging, a new strategy involving covalent grafting of ACNs onto nanocellulose was proposed in this study. For the first time, ACNs were covalently immobilized onto dialdehyde cellulose nanofibrils (DACNFs) via an acid-catalysed nucleophilic reaction between aldehyde groups on DACNFs and active hydrogens on ACNs, yielding stable ACN–DACNF (ADNF) nanocomposites. Using this approach, ACNs were effectively anchored onto nanocellulose carriers, thereby improving their stability. The resulting ADNFs were subsequently incorporated into a gelatin matrix to prepare the smart packaging film (GE-ADNF). Although GE is a widely used biopolymer, neat GE films often suffer from high water solubility, limited mechanical strength, and poor water resistance. Cross-linking between GE and ADNFs via Schiff base reactions, involving residual aldehyde groups on ADNFs and amino groups in GE, can enhance the mechanical strength and water resistance of the GE-ADNF composite films. ADNFs were characterized, and the mechanical properties, antioxidant activity, pH responsiveness, and packaging application of the GE-ADNF films were assessed to evaluate their potential as an active and smart packaging material for pork freshness monitoring.

## 2 Materials and methods

### 2.1 Materials

Fresh pork was obtained from a local market in Jinan, China. GE was supplied by Zibo Baoen Biotechnology Co., Ltd. Cellulose nanofibrils (CNFs), with a diameter of 5–10 nm and a length of 5–10 μm, were derived from bleached softwood kraft pulp, and were purchased from Tianjin Woodelf Biotechnology Co., Ltd. Sodium periodate, ammonia solution, magnesium oxide, blueberry ACN extracts with a total ACN content of 4.69%, boric acid, methylene blue, and methyl red were purchased from Shandong Keyuan Biochemical Co., Ltd.

### 2.2 Preparation of ADNFs

Preparation of DACNFs: CNFs were oxidized using sodium periodate according to a previously reported method (Jin et al., 2015). Briefly, 1 g of CNF (on an absolutely dry basis) was mixed with 25 mL of HAc-NaAc buffer solution (pH 3.5) and 6 mmol/g of NaIO₄. The mixture was allowed to react under dark at 45 °C for 48 h. Then, 20 mL of ethylene glycol was added to terminate the reaction. The oxidized CNC solution was then transferred into a dialysis bag (MWCO:12000–14,000) and dialyzed until neutral to obtain a DACNF suspension.

Synthesis of ADNFs: Solutions with different amounts of blueberry ACNs were added dropwise to a DACNF suspension with an oven-dry weight of 1 g. The mixture was stirred at 30 °C for 10 h. After the reaction, the product was dialyzed using a 12 kDa cellulose membrane against deionized water until no free ACNs were detected via ultraviolet–visible (UV–vis) spectroscopy. Three ADNF nanocomposites with DACNF: ACN extract mass ratios of 1:1, 1:3, and 1:5 were prepared and named ADNF-1, ADNF-3, and ADNF-5, respectively. The ACN loading in ADNF was determined from the absorbance difference at 279 nm (measured on a Purkinje TU-1810 UV–vis spectrophotometer) before and after the reaction.

The total ACN content in the blueberry ACN extracts was determined according to the literature (Yun et al., 2025) using the pH differential method. Briefly, blueberry ACN extracts were dissolved in buffer solutions at pH 1.0 and 4.5. Then, the absorbance values at 520 and 700 nm was measured. The total ACN content was calculated using the following equation:

(1) $$\mathrm{Total}\;\mathrm{ACN}\;\mathrm{content}\;\left(\mathrm{mg}/g\right)=\frac{\mathrm{\Delta A}\times M_{w}\times \mathrm{DF}\times 1000\times V}{\varepsilon \times l\times W}$$

where *ΔA* represents the difference between (A_520_–A_700_) at pH 1.0 and (A_520_–A_700_) at pH 4.5, *M*_*W*_ is the molecular weight of cyanidin-3-O-glucoside (449.2 g/mol), DF is the dilution factor, 1000 is the factor for converting g into mg, *V* is the volume of the solution (L), *ε* is the molar extinction coefficient of cyanidin-3-O-glucoside (26,900 L/mol·cm), *l* is the path length of the cuvette (1 cm) and *W* is the weight of blueberry anthocyanin extracts (g). The total ACN content of the blueberry ACN extracts was determined to be 46.9 mg/g.

The total ACN content in ADNFs was calculated by multiplying the ACN loading in ADNFs with 46.9 mg/g.

### 2.3 Preparation of GE-ADNF films

The GE-ADNF films were prepared via solution casting. First, a 10 wt% GE solution was prepared as the matrix. ADNFs were then incorporated into the solution at loadings of 5, 10, 15, 20, and 25 wt% relative to the GE mass. The mixtures were stirred continuously at 50 °C for 30 min in a water bath. The uniform film-forming solutions were then poured into Petri dishes and dried under controlled conditions (25 °C, 50% relative humidity (RH)) to obtain solid films, which were denoted as GE-ADNF5%, GE-ADNF10%, GE-ADNF15%, GE-ADNF20%, and GE-ADNF25%, respectively.

### 2.4 Characterization and property measurement

#### 2.4.1 Fourier transform infrared (FT-IR) spectroscopy

FT-IR spectra of the ADNFs and GE-ADNF films were recorded on an IR Prestige-21 spectrometer (Shimadzu, Japan) in attenuated total reflectance (ATR) and pellet modes, respectively. All samples were scanned over a wavenumber range of 4000–400 cm^−1^ at a resolution of 16 cm^−1^.

#### 2.4.2 X-ray diffraction (XRD)

XRD analyses of freeze-dried ADNFs and GE-ADNF films were performed on a D8 Advance diffractometer (Bruker, Germany) equipped with a Cu Kα radiation source (40 kV, 30 mA). The measurements were conducted over a 2θ range of 5°–80° at a scan rate of 2°/min.

#### 2.4.3 Atomic force microscopy (AFM)

The surface morphologies of CNF, DACNF, and ADNF were characterized using a Multimode 8 atomic force microscope (Bruker, Germany). Diluted samples were deposited onto freshly cleaved mica sheets, air-dried, and imaged in the tapping mode.

#### 2.4.4 Thermogravimetric analysis (TGA)

The thermal stability of the films was assessed via TGA using a Mettler TGA-2 instrument (Mettler-Toledo, Switzerland). Samples (∼10 mg) were heated from room temperature to 600 °C at 10 °C/min under a nitrogen atmosphere (20 mL/min).

#### 2.4.5 Scanning electron microscopy (SEM)

GE-ADNF films were cut to suitable dimensions, fixed on a sample holder using conductive tape, and sputter-coated with gold for 100 s. The surface and cross-sectional morphologies were then examined using a Hitachi Regulus 8220 scanning electron microscope (Hitachi High-Tech Corporation, Japan).

#### 2.4.6 Mechanical properties

The film thickness was measured at five randomly selected points using a digital micrometer (Syntek Co., Ltd., China), and the mean value was recorded. The films were then cut into dumbbell-shaped specimens with a gauge length of 18 mm and a width of 3.5 mm. Tensile testing was conducted using a TA.TX PlusC texture analyzer (Stable Micro Systems Ltd., UK) at a crosshead speed of 20 mm/min. For wet strength evaluation, specimens were soaked in water for 1 h and then tested under the same conditions. The Young's modulus (YM) was obtained by linearly fitting the initial elastic portion of the stress–strain curve using Origin 2018 software. The toughness (TN) was then calculated by integrating the area beneath the stress–strain curve from the origin to the fracture point. All measurements were performed in at least triplicate.

#### 2.4.7 Water contact angle (WCA) measurements

The WCA of the films was determined using an OCA50 goniometer (DataPhysics, Germany). A water droplet (2 μL) was first placed onto the film surface using a microsyringe. The static contact angle was measured, and the average of three independent measurements for each sample is given as the result.

#### 2.4.8 Water vapor transmission rate (WVTR) measurements

The WVTR of the films was measured using a W3/060 water vapor transmission rate tester (Labthink Instrument Co. Ltd., China) at 25 °C and 90% RH. Each sample was tested at least three times to ensure accuracy.

#### 2.4.9 Film swelling ratio (SR) and water solubility (WS)

The films were cut into strips (10 mm × 20 mm) and dried at 45 °C for 24 h to obtain the initial dry weight (w1). The samples were then immersed in deionized water for the following time intervals: 0.5, 1, 3, 6, and 24 h. After each immersion period, the strips were removed, gently blotted with filter paper to eliminate surface water, and weighed to obtain the wet weight (*w₂*). After 24 h immersion, the samples were re-dried at 45 °C until constant weight, which was recorded as the final dry weight (*w₃*). The swelling ratio and water solubility were calculated using the following equations:

(2) $$\mathrm{SR}\left(\%\right)=100\left(w_{2}-w_{1}\right)/w_{1}$$

(3) $$\mathrm{WS}\left(\%\right)=100\left(w_{1}-w_{3}\right)/w_{1}$$

Each sample was tested at least three times to ensure accuracy.

#### 2.4.10 UV–vis spectra

The UV–vis transmittance spectra of the films were measured using a Shimadzu UV-2600 spectrophotometer over a wavelength range of 200–800 nm.

#### 2.4.11 Antioxidant activity

The antioxidant activity of the samples was assessed using the DPPH method as described in the literature (Guo, Xu, et al., 2024). Film samples (20 mg) were subjected to reaction in a 0.2 mmol/L DPPH–methanol solution in the dark at room temperature. After 1 and 12 h of reaction, the films were withdrawn. The absorbance of the solution was measured at 517 nm on a Purkinje TU-1810 UV–vis spectrophotometer, using a film-free DPPH–methanol solution as the control. The DPPH radical scavenging rate was calculated using the following equation:

(4) $$\text{DPPH inhibition rate}\;\left(\%\right)=100\left(A_{0}-A_{1}\right)/A_{0}$$

where *A₀* and *A*_*1*_ are the absorbances of the control and films at 517 nm.

To ensure data reliability, each sample was subjected to at least three independent tests.

#### 2.4.12 ACN release performance

To determine the ACN release profile, film samples (20 mm × 20 mm) were weighed and immersed in 100 mL of deionized water in a 250 mL conical flask. The flask was maintained at 25 °C while shaking at 150 rpm. At predetermined intervals, 3 mL of the supernatant was collected and immediately replaced with an equal volume of fresh deionized water to keep the total volume constant. After centrifugation, the absorbance of the supernatant at 279 nm was measured using a Purkinje TU-1810 UV–vis spectrophotometer. The cumulative release of ACNs was calculated from the absorbance values using the standard curve for blueberry ACN extracts. To ensure data reliability, each sample was subjected to at least three independent tests.

#### 2.4.13 Color response of ACNs, ADNFs and GE-ADNF films at different pH

The pH-responsive color changes of the ACN solution and ADNF suspension were examined by adjusting the pH from 2 to 10 using buffers and photographing the results. For the films, color was quantified using an RM200QC colorimeter (X-Rite Inc., USA) in the CIE-L*a*b* system (mean of three measurements). Film samples (20 mm × 20 mm) were then immersed in pH 2–10 buffers for 30 min, and the color was measured again after surface drying to calculate ΔE using the following equation:

(5) $$\mathrm{\Delta E}=\sqrt{{\left(L^{*}-{L_{0}}^{*}\right)}^{2}+{\left(a^{*}-{a_{0}}^{*}\right)}^{2}+{\left(b^{*}-{b_{0}}^{*}\right)}^{2}}$$

where L*, a*, and b* are the color parameters of the sample after immersion into the pH buffer solutions, and *L*_0_*, *a*_0_*, and *b*_0_* are the color parameters of the sample before immersion.

#### 2.4.14 Color stability of GE-ADNF films

The GE-ADNF films were stored in a constant temperature and humidity chamber at 60 °C with 50% RH for a certain period of time. The color of the films was recorded at intervals using a color difference meter, and the ΔE was calculated using Eq. (5), where *L*_0_*, *a*_0_*, and *b*_0_* are the color parameters of the initial film, and L*, a*, and b* are the color parameters of the film after storage. All measurements were performed in at least triplicate.

### 2.5 Application in pork freshness detection

#### 2.5.1 Ammonia response of GE-ADNF films

Film samples (30 mm × 40 mm) were placed 1 cm above a 25%–28% ammonia solution. Photographs were taken at specific intervals to record the color transition, and color parameters were determined using an RM200QC colorimeter (X-Rite, US). The resulting ΔE was calculated using Eq. (5), where *L*_0_*, *a*_0_*, *b*_0_*, and L*, a*, b* represent the color parameters of the initial film and the film exposed to ammonia solution for a certain period of time.

#### 2.5.2 Application of GE-ADNF films in pork freshness detection

Fresh pork tenderloin (16.5 g) was packaged in a plastic Petri dish, with the film fixed to the inner surface of the lid. Over time, changes in film color and the total volatile basic nitrogen (TVB-N) level of the pork sample were monitored at 6- or 12- h intervals. TVB-N and pH were determined using the Kjeldahl method according to the literature (Qin et al., 2019). Film color were measured periodically, and the Δ*E* was calculated using Eq. (5), where *L*_0_*, *a*_0_*, *b*_0_*, and L*, a*, b* represent the film's initial and time dependent color parameters, respectively.

### 2.6 Statistical analysis

Data were analyzed using SPSS (SPSS Inc., Chicago, USA) and Origin 2018. To compare means and assess significant differences, Duncan's multiple range test was used with a threshold of *p* < 0.05.

## 3 Results and discussions

### 3.1 Preparation and characterization of ADNF nanocomposites

**Scheme 1:**
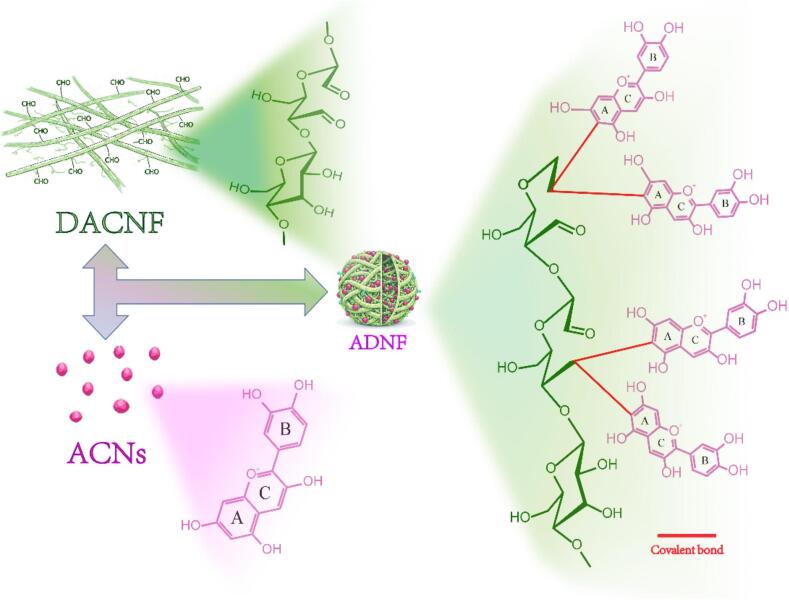
Schematic preparation route of ADNFs

The ANDF nanocomposites were prepared using a two-step procedure, as outlined in Scheme 1. CNFs were first periodate-oxidized to DACNFs. Subsequently, blueberry ACNs were covalently immobilized onto the DACNFs via an acidic nucleophilic reaction between aldehyde groups on the DACNFs and active hydrogens on ACNs (Yun et al., 2025), producing anthocyanin–DACNF (ADNF) nanocomposites. As shown in Scheme 1, under acidic conditions, the aldehyde groups are activated and attack the active hydrogen at C-6 or C-8 positions on the A-ring of ACNs, substituting them to form a stable covalent bond (Yun et al., 2026), while the phenolic hydroxyl groups responsible for pH responsiveness remained preserved. The covalent grafting was expected to enhance the stability of ACNs.

AFM was used to examine the morphology of the ADNF nanocomposite. As expected for nanocellulose, the CNFs and DACNFs exhibited fibrillar structures with nanoscale diameters and high aspect ratios. After the reaction with ACNs, a pronounced morphological change was observed, with the ADNFs assembling into ellipsoidal nanoparticles, confirming the successful reaction between ACNs and the DACNFs. During this process, ACNs acted as a cross-linking agent, driving the reorganization of the nanofibrils into nanocomposite particles. ADNF1, with the lowest ACN dosage, showed the largest particle diameter together with some non-crosslinked nanofibrils. As the ACN dosage increased, these residual nanofibrils were no longer observed in ADNF3 and ADNF5, suggesting an increased crosslinking density. ACNs were covalently bonded onto the nanofibrils to form spherical nanocomplexes, and therefore improved stability was expected compared to free ACNs.

**Fig. 1:**
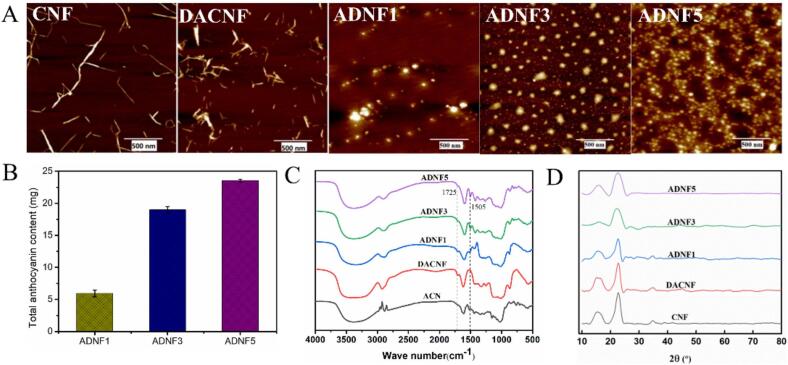
A, AFM images of CNF, DACNF, ADNF1, ADNF3 and ADNF5; B, ACN content in ADNF nanocoposites; C, FT-IR and D, XRD patterns of CNFs, DACNFs and ADNFs.

The total ACN content in the ADNFs was determined using the pH differential method. As shown in Fig. 1B, ACN contents of 5.94, 18.98, and 23.54 mg were obtained for ADNF1, ADNF3, and ADNF5, respectively, indicating that a higher ACN dosage led to an increased ACN content in the ADNF nanocomposite. The ACN content in ADNF3 and ADNF5 was higher than that of purple sweet potato anthocyanin grafted onto dialdehyde locust bean gum (PSPA-g-DALBG), which has a content of 13 mg/g (Yun et al., 2025). Compared with the FT-IR spectrum of the CNFs, those of DACNF showed a characteristic carbonyl absorption peak at 1725 cm^−1^ (Fig. 1C), confirming the successful introduction of aldehyde groups. After the reaction with ACNs, the intensity of this peak decreased, indicating that the aldehyde groups in DACNFs participated in the reaction with ACNs. Nevertheless, this peak was still observed in the spectra of all ADNFs, suggesting that residual, unreacted aldehydes remained. The characteristic peak at 1505 cm^−1^ in the ACN spectrum, ascribable to the C=C stretching vibration of aromatic rings, was also detected in the spectra of the ADNFs. This provides direct evidence for successful incorporation of ACN into the nanofibrils. The XRD patterns of the CNFs, DACNFs, and ADNFs all showed characteristic peaks at 2θ values of 16.0° and 22.6°, corresponding to the (110) and (200) planes of cellulose I, respectively. This indicates that neither periodate oxidation nor ACN conjugation disrupted the fundamental cellulose I crystalline structure. The observed decrease in peak intensity for all the ADNFs can be attributed to the amorphous nature of the incorporated ACNs.

Both the ACN solution and ADNF5 suspension exhibited clear color changes across a pH range of 2–10 (Fig. S1). The color shifted from light red for ACNs or light brown for ADNF under acidic conditions (pH 2–6), to deep brown at neutral pH 7, and finally to brownish-blue at alkaline pH (8–10). This strong pH responsiveness, resulting from structural transformations of the ACN chromophore, confirms that covalent grafting of ACNs onto the nanofibrils does not impair their inherent pH-sensitive property. The phenolic hydroxyl groups of ACNs were not involved in the reaction with nanocellulose and therefore did not influence its color-changing behavior.

### 3.2 Preparation and characterization of GE-ADNF films

**Scheme 2:**
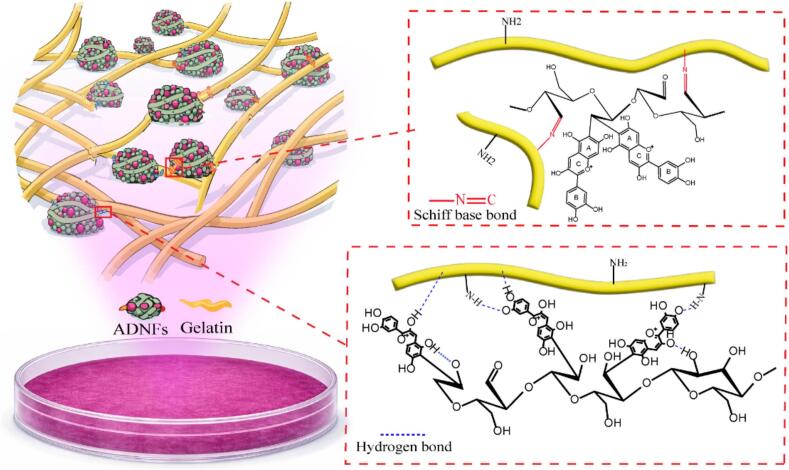
Interactions between gelatin and ADNFs.

Since ADNF5 showed the highest ACN content and a highly uniform morphology, GE-ADNF films with different ADNF percentages were prepared using GE as the matrix and ADNF5 as the nanofiller. As shown in Scheme 2, the GE-ADNF films were formed through multiple interactions between GE and ADNFs. The amino groups in GE reacted with residual aldehyde groups on ADNFs to form imine bonds (—C=N) through a Schiff base reaction. Hydrogen bonding among GE, CNFs, and ACNs also contributed to the formation of the composite films.

**Fig. 2:**
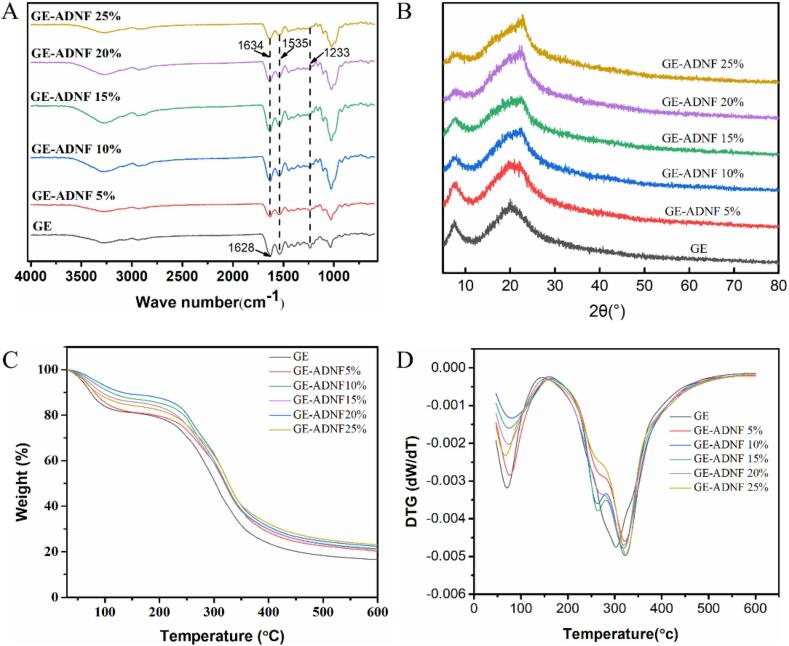
A. FT-IR spectra; B, XRD patterns; C, TG; and D, DTG of the composite films.

Fig. 2A presents the FT-IR spectra of GE and the GE-ADNF films. The peaks at 1629, 1535, 1233 cm^−1^ were assigned to amides I, II, and III in GE, respectively. After the incorporation of ADNFs, the amide I peak shifted to 1634 cm^−1^, which may be attributed to the interaction with the hydroxyl group of nanocellulose. Compared with the spectra of ADNF (Fig. 1C), the band at 1725 cm^−1^, corresponding to the —C=O group, disappeared in all GE0ADNF films, confirming the occurrence of the Schiff base reaction between GE and ADNFs. Unexpectedly, no band between 1650 and 1600 cm^−1^ corresponding to the imine bond (—C=N) was observed, likely because the signal overlapped with (or was obscured by) the amide I band of GE and the hydroxyl band of cellulose. The XRD patterns of the films are presented in Fig. 2B. ACNs from different sources are amorphous (Koosha & Hamedi, 2019), and the XRD patterns of ACN-rich biopolymer-based films largely retain the original diffraction peaks (Mohammadalinejhad et al., 2020). The neat GE film exhibited two characteristic peaks: a peak at 2θ = 20°, attributable to its amorphous phase (Mosleh et al., 2021), and a peak at 2θ = 7.5°, corresponding to the crystalline triple-helix structures of GE. In the XRD patterns of GE-ADNF films, the broad amorphous peak shifted to 22°–22.6°, which overlapped with that of the (002) plane of cellulose I. As the ADNF content increased, the intensity of this peak increased, whereas the peak at 7.5° gradually decreased. This reduction may be attributed to the formation of hydrogen bonds between the hydroxyl groups of ACNs and CNFs and amino groups of GE, which likely suppressed the formation of triple-helix structures (Mosleh et al., 2021).

The thermal degradation behavior of the GE and GE-ADNF films was evaluated via TGA and DTG, as shown in Fig. 2C and D. The neat GE film exhibited a two-stage weight loss: the first stage (below 100 °C) corresponded to the evaporation of free water, and the second major stage (200 °C–400 °C) was associated with the degradation of peptide bonds and triple-helix structures (Mosleh et al., 2021). In contrast, the GE-ADNF films exhibited three distinct degradation stages: a first one associated with water evaporation, a second one due to the ACN decomposition, and a third one corresponding to the concurrent breakdown of the GE and cellulose matrix. The incorporation of ADNF considerably improved the thermal stability of the films, as reflected by a higher residual weight after decomposition (Fig. 2C) and a shift in the maximum weight loss rate toward higher temperatures (Fig. 2D). For example, GE-ADNF25% showed the highest residual weight of 23.06%, whereas the neat GE film exhibited a residual weight of 16.70% for, which is consistent with the higher ADNF content in the former film. The improved thermal resistance of the GE-ADNF films can be explained by two main factors. First, the polyphenolic structure of ACNs enhances the thermal stability of biopolymers (Eze et al., 2022; Jiang et al., 2020). Second, the strong covalent and hydrogen bonds within the film matrix require additional thermal energy to dissociate (Jiang et al., 2023). Previous studies have indicated that incorporating ACN-rich extracts effectively enhances the thermal stability of polymeric films (Eze et al., 2022; Jiang et al., 2023). The increase in the first major degradation temperature (T₁) also suggests that water evaporation was restricted in the GE0ADNF films. This slower moisture loss is associated with reduced material brittleness (Lin et al., 2020), which is beneficial for practical applications.

**Fig. 3:**
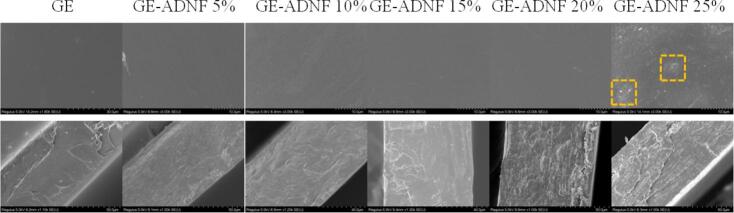
Surface (upper row) and cross-section (lower row) SEM images of GE and GE-ADNF films.

SEM images (Fig. 3) showed that incorporation of ADNFs noticeably affected the morphology of the GE films. The neat GE film exhibited a smooth and uniform structure, which is attributable to the good film-forming ability of GE. Notably, GE-ADNF films with ADNF content up to 20% retained this smooth morphology, suggesting good compatibility between the two components. This compatibility is important for overall film performance. At a concentration of 1%, ACNs disrupted the dense molecular structure of the film, thereby causing heterogeneous dispersion of the film-forming matrix (Zhang et al., 2020). The high compatibility in this system, even at a filler loading of 20%, was likely due to the homogeneous shape of ADNFs (Fig. 1a), and strong Schiff base and hydrogen bonds within the GE matrix. Zhang et al. (2020) also reported that the uniform dispersion of ACN-loaded nanocomposites within the polymer matrix allowed them to function as effective compatibilizers for starch and polyvinyl alcohol (PVA). Clear morphological changes were observed at 25% ADNF loading (GE-ADNC25%), with the corresponding GE-ADNF25% exhibiting increased surface roughness and filler aggregation (marked in yellow).

### 3.3 Mechanical properties

**Fig. 4:**
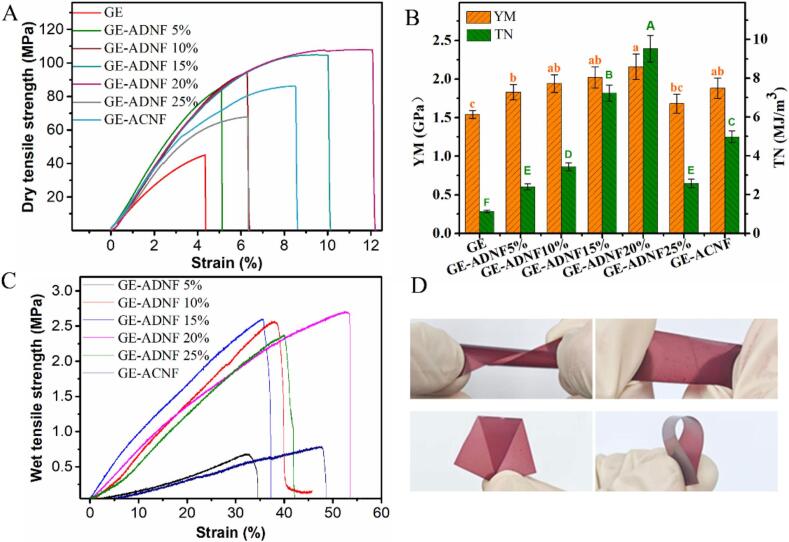
A, Dry tensile strength; B, Young's modulus (YM) and toughness (TN); C, wet tensile strength of GE and GE-ADNF films; D, folding and stretching of GE-ADNF20%. Different letters on the bars indicated significant difference of the mean values (*p* < 0.05).

Adequate mechanical properties are necessary for food packaging films to maintain food integrity during transportation and handling. The dry and wet tensile strength and strain of the films are presented in Fig. 4A and B. After ADNF incorporation, both the tensile strength and strain of the GE-ADNF films increased markedly. For example, the tensile strength and strain of the neat GE film were 45.33 MPa and 4.33%, respectively, whereas those of the film with 5% ADNF addition increased by 88.20%, 85.31 MPa, and by 17.79%, 5.10%. Both stress and strain continued to increase until the ADNF content reached 20%. The maximum tensile strength and strain were obtained at 20% ADNF addition, reaching 107.84 MPa and 12.08%, respectively. However, when the ADNF content increased to 25%, the film showed a clear decline in mechanical performance, which may be related to the non-uniform film morphology (Fig. 3). The improvement in tensile strength may be explained by several factors, including the reinforcing effect of nanofillers, covalent bonding between GE and ADNFs, and stronger hydrogen bonding among GE, nanocellulose, and ACNs. The increased number of dynamic Schiff base bonds and hydrogen bonds within the network also acted as sacrificial bonds, helping dissipate energy and improve strain tolerance. The addition of ACNs also enhances elongation at break owning to the plasticizing effect of ACNs (Jamróz & Kopel, 2020). These findings are consistent with the study of Liu et al. (2022), who prepared an intelligent colorimetric film by introducing ACNs encapsulated in ovalbumin-carboxymethyl cellulose (OVA-CMC) nanocomplexes into a corn starch-PVA matrix. They observed a marked increase in both tensile stress and strain after incorporating the OVA-CMC-ACN nanocomplex. The tensile strength substantially surpassed the values reported for some other GE-based ACN films (Table S1), e.g. 16.65–17.97 MPa for a GE-purple cabbage anthocyanin (PCA)-chondroitin sulphate (CS) (GE-PCA-CS) film (Hao, Pang, Mraz, Geng, Liu & Pan, 2024), and 30 MPa for a bacterial cellulose (BC)/GE/fluorescein isothiocyanate (FITC)/red cabbage (PCA) film (Yang et al., 2024). To examine the influence of covalently bonding ACNs to nanofibrils, a gelatin-ACN-CNF (GE-ACNF) film was prepared containing the same amounts of CNFs without periodate oxidation and ACNs as in the 20% ADNF5 nanocomposite. The GE-ADNF film exhibited higher tensile strength, which can be attributed to Schiff base bonding between DACNFs and GE, as well as improved interfacial compatibility of the spherical nanofillers within the GE matrix. The incorporation of ADNFs increased both the YM and TN of the GE-ADNF films (Fig. 4B), with GE-ADNF20% reaching the highest values. The film also showed good foldability and stretchability (Fig. 4D). This balance of improved strength and flexibility is beneficial for packaging applications.

**Fig. 5:**
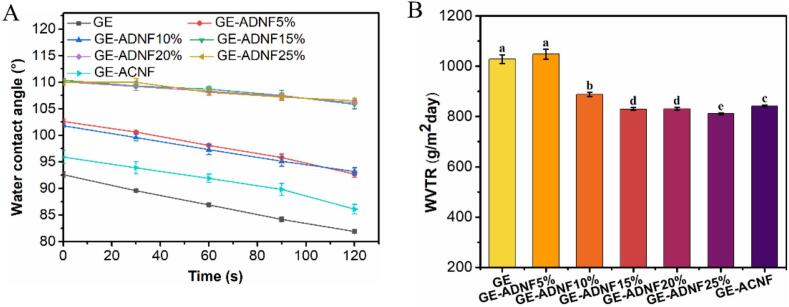
A, WCA, and B, WVTR of the GE and GE-ADNF films. Different letters on the bars indicate significant differences in the mean values (*p* < 0.05).

The wet tensile properties of the films were measured after 1 h of water immersion to evaluate their performance under high-humidity conditions. The neat GE film nearly dissolved, while all GE-ADNF films remained intact. Fig. 5b indicates that the wet GE-ADNF films showed notably higher elongation at break than the dry films, owing to the plasticizing effect of water (Li et al., 2024). Although the tensile strength decreased substantially for all films in the wet state, the GE-ADNF films (10%–20% loading) retained a tensile strength of 2–2.7 MPa. The covalent bonds in the GE-ADNF films conferred improved water stability, resulting in higher wet tensile strength compared with the GE-ACNF film.

### 3.4 WCA and water vapor barrier properties

High hydrophilicity in films is generally detrimental to their application in high-humidity environments. WCA measurements were used to assess the hydrophilicity and surface wettability of the GE-ADNF films. As shown in Fig. 5a, changes in the WCA of the GE and GE-ADNF films were monitored over 120 s. Compared with the GE film, all GE-ADNF films showed a considerable increase in WCA. This improved hydrophobicity may be attributed to two main factors. First, the covalent bonds between aldehyde groups in the ADNFs and amino groups in GE are more stable in aqueous environments, thereby increasing the WCA of the GE-ADNF films. Second, the hydrophobic moieties of ACNs exposed at the air–film interface likely contribute to the enhancing surface hydrophobicity (Li et al., 2024). As the ADNFs content increased, the WCA increased. With time, the WCA of all films gradually decreased. However, this reduction was slower in GE-ADNF films than in the GE film, likely due to covalent bonding within the films. This trend was especially obvious for GE-ADNF15%, GE-ADNF20%, and GE-ADNF25%, where the WCA decreased only from about 110° to 106° over 120 s. Notably, GE-ACNF films lacking aldehyde groups exhibited a much lower WCA than the GE-ADNF films. This further confirms the positive role of covalent cross-linking in enhancing the water-barrier properties of the films. The GE-ADNF films exhibited a higher water contact angle (∼110°) than that of the gelatin/EC/PSPA film (81.62°, Wen et al., 2024) and the BC/GE/FITC/PCA film (82.73°, Yang et al., 2024) owning to the covalent cross-linking. The enhanced hydrophobicity is conducive to their application in high-humidity environments.

The water vapor barrier properties of the GE and GE-ADNF films were assessed by measuring the WVTR, as shown in Fig. 5B. Although the incorporation of ADNFs caused a slight increase in WVTR for GE-ADNF5%, further increasing ADNF content led to lower WVTR. An increase in covalent and hydrogen bonding within the films could raise the cross-linking density, reducing the matrix free volume and extending the tortuous pathway for water vapor, thereby lowering its diffusion rate (Alizadeh-Sani et al., 2021; Yong et al., 2019). The presence of ACN also improves barrier performance, as the bulky aromatic and pyrylium rings in the ACN skeleton physically hinder the internal network of the film and further restrict water vapor transport (Kurek et al., 2018). Notably, GE-ACNF (film containing free ACNs) showed a higher WVTR than GE-ADNF20%, indicating that immobilizing ACNs on nanofibrils effectively improved the water vapor barrier. This finding aligns with previous study reporting that nanocomposite films with bound ACNs exhibited better barrier properties than those containing free ACNs (Balti et al., 2017).

### 3.5 UV-shielding, antioxidant and antibacterial properties

**Fig. 6:**
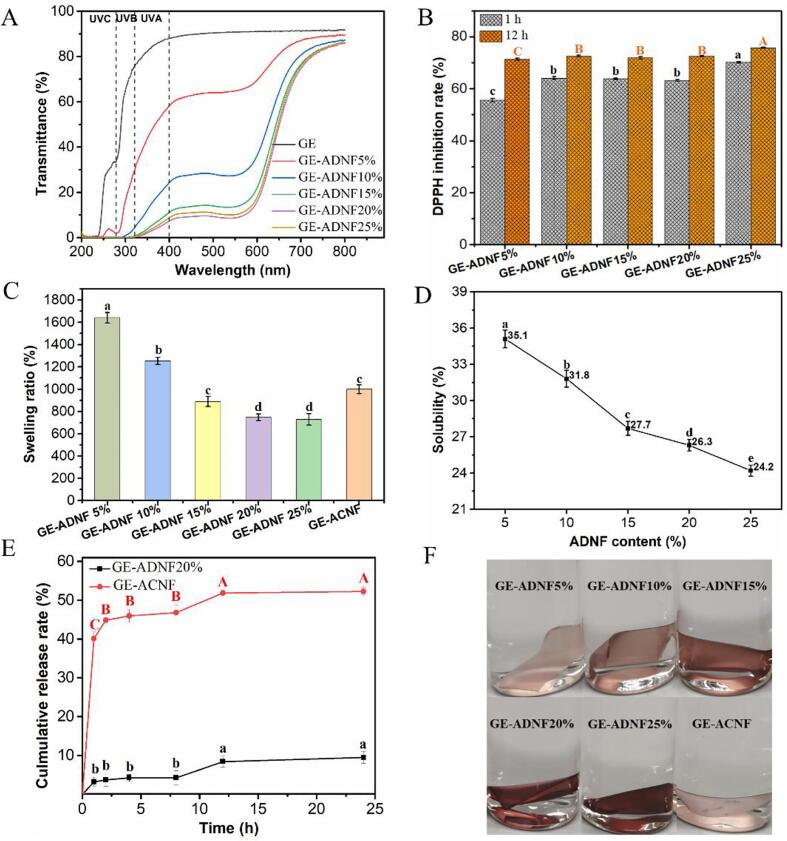
A, UV–vis spectra; B, DPPH inhibition rate; C, swelling ratio after 24 h; D, solubility; E, ACN release rate of the GE-ADNF films, and F, photographs of films in water after 24 h. Different letters indicate significant differences (*p* < 0.05).

UV radiation can reduce the nutritional value and flavor of food and promote lipid oxidation. Therefore, packaging materials with UV-shielding properties are highly desirable. ACNs show strong UV-shielding capability because the abundant aromatic rings in their structure can absorb UV radiation. The UV-shielding performance of the films was evaluated using transmittance measurements (Fig. 6A). The results indicate that incorporation of ADNF markedly reduced UV transmittance, particularly in the UVB and UVC regions, demonstrating effective UV shielding ability. This effect showed a direct relationship with the ACN loading, with higher content producing stronger UV blockage.

Unsaturated fatty acids in food are susceptible to oxidation induced by temperature, oxygen, and moisture, which accelerates food spoilage and quality deterioration while posing potential safety risks (de Oliveira et al., 2018). The antioxidant activity of the GE-ADNF films was evaluated by measuring DPPH radical scavenging rates (Fig. 6B). The GE film exhibited a DPPH inhibition rate of 9.418%, whereas the incorporation of ADNFs significantly increased the scavenging activity (p < 0.05), with all GE-ADNF films exceeding 50% after 1 h of reaction with DPPH. Since GE itself has limited antioxidant capacity, this enhancement was mainly attributed to ACNs. As natural flavonoids, ACNs contain abundant phenolic hydroxyl groups that can efficiently scavenge DPPH radicals. Accordingly, the antioxidant activity increased with increasing ADNF content. The free radical scavenging rate of all samples exceeded 70% even after 12 h, indicating sustained antioxidant effect. This prolonged activity is favorable for food packaging applications. The antioxidant activity of the composite films was lower than that of the natural antioxidant vitamin C, which achieved 100% DPPH inhibition at a low concentration (Ametal & Singh, 2014), but higher than that of BC/GE/FITC/PCA (19.08%, Yang et al., 2024) and GE-PCA-CS (53%, Hao et al., 2024).

The antibacterial activities of the GE and GE-ADNF films against *S. aureus* were evaluated using the plate count method, and the results were shown in Fig. S2. The neat gelatin film exhibited no antibacterial properties; in contrast, the GE-ADNF films demonstrated pronounced antibacterial activity, which increased progressively with rising ADNF content. Notably, the GE-ADNF20% achieved nearly 100% antibacterial activity. This remarkable efficacy is primarily attributed to the ability of blueberry ACNs to disrupt bacterial cell structures and interfere with metabolic processes (Sun et al., 2018).

Overall, the GE-ADNF films exhibited excellent UV-shielding, antioxidant and antibacterial properties, making them promising candidates for active packaging applications.

### 3.6 SR, WS and ACN release

Since the neat GE film disintegrated within 1 h in water, the swelling ratios were only measured for the GE-ADNF films (Fig. 6C and Fig. S3). Incorporation of ADNFs significantly reduced the swelling ratio, with higher ADNF loading resulting in a more pronounced reduction. This behavior can be attributed to the cross-linking effect of ADNFs, which formed strong covalent and hydrogen bonds with the GE matrix, thereby limiting water uptake and swelling. Water solubility is an important indicator of film water resistance and structural stability. Although the neat gelatin film showed a WS of 100%, dissolving completely after 24 h, the GE-ADNF films exhibited substantially decreased WS (Fig. 6D). All GE-ADNF films remained intact after 24-h immersion (Fig. 6F), confirming that water resistance was markedly improved upon ADNF incorporation. Notably, WS decreased with increasing ADNF loading. This observation is notable because ACNs are generally hydrophilic and often increase film WS when added in free form (Zhang et al., 2019). The decrease observed here may result from the formation of a dense cross-linked network through covalent and hydrogen bonding between ADNFs and GE, which limits water penetration and suppresses polymer dissolution. This agrees with the results reported by Amaregouda et al. (2022), who found that increased anthocyanin content in chitosan/PVA films reduced WS because ACNs interacted with polymer chains and masked hydrophilic hydroxyl groups. As shown in Fig. 6E, the ACN release rate of GE-ADNF20% was substantially lower than that of the GE-ACNF film, owing to the stable covalent bonding between ACNs and DACNF. Yun et al. (2025) reported that grafting PSPA onto DALBG also reduced the release of ACNs. This reduced release is particularly beneficial for application as indicator films under high-humidity conditions.

### 3.7 pH responsiveness and color stability of the GE-ADNF films

The L*, a*, and b* values of the GE-ADNF films were measured under different pH conditions, and the corresponding ΔE was calculated (Table S2). In the CIE L*a*b* system, the a* coordinate represents the green (−) to red (+) axis, while the b* coordinate represents the blue (−) to yellow (+) axis. As pH increased, the a* value decreased for all films, indicating an obvious color shift from intense redness under acidic conditions to a greenish hue in alkaline environments. This pattern was consistent with the visual appearance of the films across the tested pH range. Similarly, the b* value also decreased across the pH range of 2–10. The ΔE was used to quantify the colorimetric variation of the indicator films. According to Huang, Du, et al. (2023) and Huang, Tsai, et al. (2023), color changes become visually observable when ΔE exceeds 5. The highest ΔE values were recorded between pH 6 and 10, indicating pronounced color transitions under slightly acidic to basic conditions. These results are consistent with those reported by Huang, Du, et al. (2023) and Huang, Tsai, et al. (2023). Covalent grafting did not block the phenolic hydroxyls essential for pH-induced chromophore interconversion, thus preserving full pH-responsiveness.

**Fig. 7:**
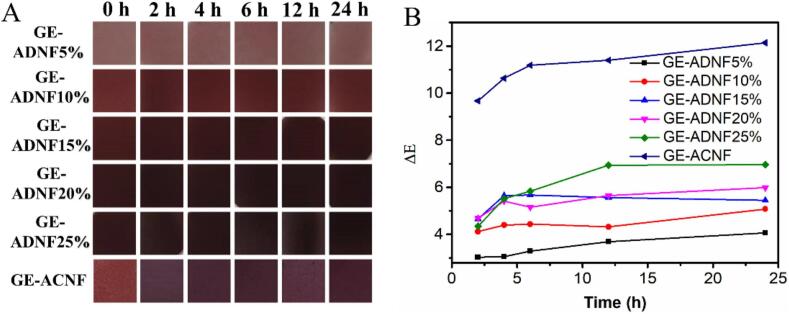
Color stability of the GE-ADNF films at 60 °C.

ACNs are highly susceptible to degradation when exposed to heat, light, and oxygen, which considerably limits their application in intelligent food packaging. Among these factors, heat is considered the most damaging to ACN stability (Yun et al., 2025). As shown in Fig. 7, the color stability of the GE-ADNF films was examined at 60 °C. All films exhibited color changes due to thermal degradation of ACNs (Huang et al., 2021). However, compared with the GE-ACNF film, all GE-ADNF films showed markedly improved color stability. The ΔE value of the GE-ACNF reached 13 after 24 h, whereas those of all GE-ADNF films remained within 5–7. In the GE-ADNF films, higher ADNF content resulted in greater color changes, which may be attributed to increased ACN loading. Notably, after 24 h, the ΔE values of GE-ADNF5%, GE-ADNF10%, and GE-ADNF15% remained below 5, indicating good color stability. The strong covalent bonds anchors ACNs onto the DACNFs, effectively preventing the aggregation, detachment, or structural rearrangement of ACN molecules upon heating, thereby considerably enhancing their resistance to heat-induced degradation (Yun et al., 2026). Previous studies have also shown that other flavonoid-grafted dialdehyde polysaccharides exhibit higher stability than their free flavonoid counterparts (Hu et al., 2021; Yun et al., 2025).

### 3.8 Application in freshness monitoring of pork samples

**Fig. 8:**
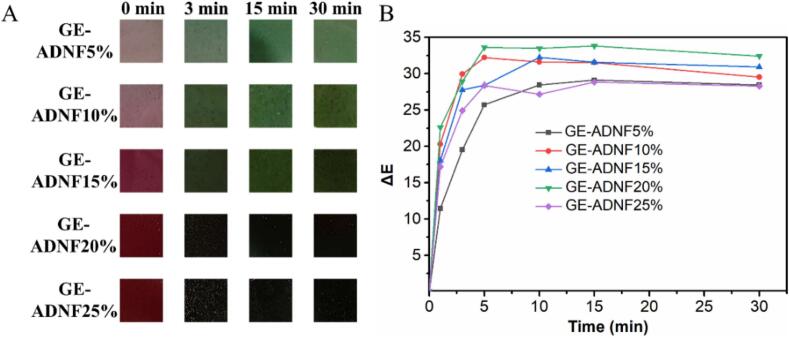
A, Color changes of films exposed to volatile ammonia; B, relationship between exposure time and color difference (ΔE) of the films.

The GE-ADNF films were exposed to volatile ammonia and photographed, and the corresponding ΔE values were calculated to evaluate their response to volatile nitrogen compounds. The results are shown in Fig. 8A and B. Within 3 min of exposure, the color of all films changed from red to light/deep green as the environment became alkaline, with ΔE values exceeding 20, well above the visual threshold of ΔE > 5. These results indicate high sensitivity of the GE-ADNF films to ammonia, consistent with those reported for ACN-incorporated bacterial cellulose (BC) films (Moradi et al. (2019), which changed from an initial deep carmine to dark blue after 4 min of ammonia exposure. Notably, the response of the present films was faster than that of starch/chitosan/roselle ACN and PVA/chitosan/roselle ACN films, which required 24 min to show an obvious color change (Zhang et al., 2019). This rapid response is highly desirable and demonstrates the potential of these films for practical application in intelligent packaging of protein-rich foods. The color change of the GE-ADNF films reached a maximum after 5–10 min of exposure and remained stable thereafter. Given that GE-ADNF10%, GE-ADNF15%, and GE-ADNF20% showed higher ΔE values than GE-ADNF5% and GE-ADNF25%, they were selected for subsequent experiments as indicators for pork freshness. To test the color reversibility, GE-ADNF20% was repeatedly and alternately exposed to the ammonia and acetic acid solutions for 3 min each (Huang, Du, et al., 2023; Huang, Tsai, et al., 2023). Images of the tested sample are shown in Fig. S4. After exposure to acetic acid, the color changed back to the initial red due to the structural transformation from the quinonoid base back to the flavylium cation, which indicates good reversibility.

**Fig. 9:**
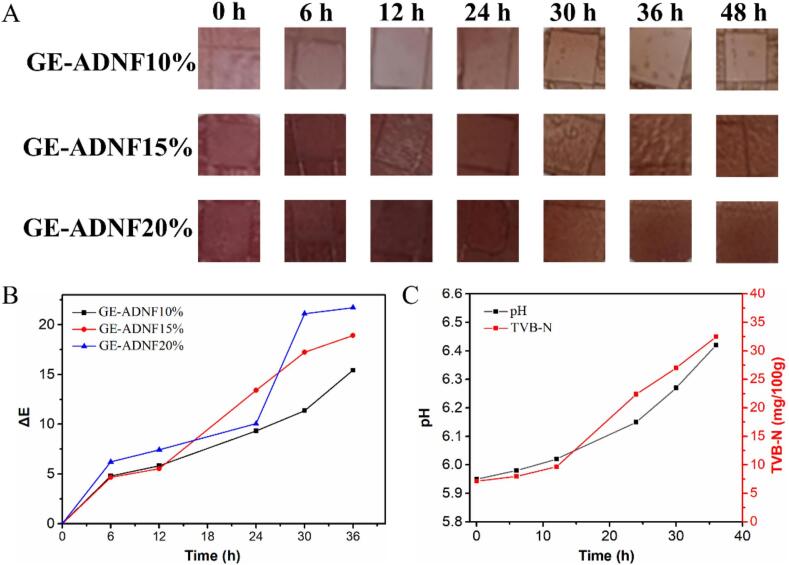
A, Photographs of films during pork storage, B, color difference (ΔE) of the films vs. preservation time, and C, pH and total volatile basic nitrogen (TVB-N) vs. time of the pork samples.

The GE-ADNF films were applied as freshness indicators for pork stored at 25 °C. As revealed by the photographs and ΔE values (Fig. 9A, B), the films displayed distinct color changes throughout storage. Proteins in meat are highly vulnerable to microbial spoilage by bacteria and mold. During this process, various volatile nitrogenous compounds, including ammonia, trimethylamine, and other amines, are produced and released, thereby triggering the color transition of the indicator films.

**Fig. 10:**
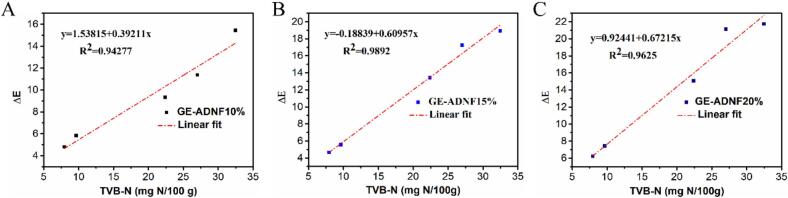
Correlation between total volatile basic nitrogen (TVB-N) and color difference (ΔE): A, GE-ADNF10%, B, GE-ADNF15% and C, GE-ADNF20%.

To objectively evaluate pork freshness, pH and TVB-N values were determined (Fig. 10C). Initial measurements on day 0 (pH: 5.95; TVB-N: 7.14) confirmed that the pork was fresh. Within the first 12 h, the TVB-N value increased to 9.66 mg N/100 g, whereas the pH remained relatively stable (6.02), indicating that the meat was still fresh. Correspondingly, the indicator films showed only slight color changes, with ΔE values between 5 and 7. After 24 h, pH and TVB-N increased to 6.15 and 22.4 mg N/100 g, respectively. The TVB-N value was close to the rejection limit for pork (25 mg N/100 g), suggesting that the meat was still edible but nearing the end of optimal freshness. At this stage, the films turned deep brown. GE-ADNF15% showed the most evident color change, with a maximum ΔE of 13.4, indicating a rapid response to freshness changes in pork. The pH change also matched established pH thresholds for pork freshness, which can be categorized as fresh (5.18–6.12), sub-fresh (6.13–6.16), and onset of spoilage (>6.17) (Choi et al., 2017). By 30 h of storage, the TVB-N value reached 27 mg N/100 g and the pH reached 6.27, confirming the spoilage of the pork samples. The films shifted to a brownish-yellow hue, with ΔE values ranging from 11 to 21, clearly visible to the naked eye. The rapid response of the film during the early stages of spoilage underscores their strong potential for practical application in real-time freshness monitoring of pork samples.

Linear fitting was performed to examine the relationship between the TVB-N values of the pork samples and the ΔE values of the films. The results indicated a strong positive correlation (Fig. 10), with R^2^ values of 0.94, 0.99, and 0.96 for the respective films. Notably, GE-ADNF15% showed the highest correlation. This result is consistent with the close relationship between TVB-N and ΔE reported by Moradi et al. (2019) in their study of common carp during storage.

## 4 Conclusions

In this study, a stable ADNF nanocomposite was successfully synthesized by covalently grafting blueberry ACNs onto periodate-oxidized cellulose nanofibrils. The ADNF was incorporated into a GE matrix to produce intelligent packaging films (GE-ADNF) with multifunctional properties through Schiff base and hydrogen bonding interactions. Compared with physical blending, covalent immobilization effectively reduced ACN leaching, improved water resistance and color stability, and retained full pH-responsiveness, outperforming non-covalent controls. The GE-ADNF films achieved a maximum tensile strength of 107.84 MPa and a strain of 12.08% at 20 wt% ADNF content, a WCA of ∼110°, near-complete UV-shielding, sustained antioxidant activity (>70% DPPH scavenging after 12 h), and excellent color stability (ΔE <5 at 60 °C for 24 h). In pork freshness monitoring at 25 °C, GE-ADNF15% showed the highest sensitivity (R^2^ = 0.99 with TVB-N), clearly distinguishing fresh, sub-fresh, and spoiled states. In terms of mechanical and sensitivity for spoilage monitoring, GE-ADNF15% represents the optimal balance. To the best of our knowledge, this is the first report on covalent immobilization of anthocyanins onto nanocellulose for intelligent food packaging applications. This work presents an effective approach for stabilizing ACNs and offers a promising, environmentally friendly material for real-time monitoring of pork freshness. The covalent strategy may be applicable to other natural pigments. The films can be used as on-package colorimetric labels for pork and other protein-rich foods. Their high wet strength and low solubility enable their use in humid environments (e.g. refrigerated meat packaging). Limitations include laboratory-scale production, higher cost due to periodate oxidation and dialysis steps, and the need for long-term validation under real supply chain conditions (freezing, light, transport stress). Future studies should evaluate the economic feasibility and environmental sustainability of large-scale DACNF production for commercial intelligent packaging applications.

## CRediT authorship contribution statement

**Jiawei Zheng:** Writing – original draft, Investigation. **Yang Zhang:** Investigation. **Xidong Li:** Investigation. **Qinghua Xu:** Writing – review & editing, Supervision, Funding acquisition. **Liqiang Jin:** Writing – review & editing, Funding acquisition.

## Funding

This research was funded by the 10.13039/501100001809National Natural Science Foundation of China [grant No. 22078165], the QUTJBZ program [grant No. 2024ZDZX01] and Major Scientific Research Project for the Construction of State Key Lab [grant No. 2025ZDGZ02].

## Declaration of competing interest

The authors declare that they have no known competing financial interests or personal relationships that could have appeared to influence the work reported in this paper.

## Data Availability

Data will be made available on request.

## References

[bb0005] Alizadeh-Sani M., Mohammadian E., Rhim J.W., Jafari S.M. (2020). pH-sensitive (halochromic) smart packaging films based on natural food colorants for the monitoring of food quality and safety. Trends in Food Science & Technology.

[bb0010] Alizadeh-Sani M., Tavassoli M., McClements D.J., Hamishehkar H. (2021). Multifunctional halochromic packaging materials: Saffron petal anthocyanin loaded chitosan nanofiber/methyl cellulose matrices. Food Hydrocolloids.

[bb0015] Amaregouda Y., Kamanna K., Gasti T. (2022). Fabrication of intelligent/active films based on chitosan/polyvinyl alcohol matrices containing Jacaranda cuspidifolia anthocyanin for real-time monitoring of fish freshness. International Journal of Biological Macromolecules.

[bb0020] Ametal R.K., Singh M. (2014). A thermodynamic in vitro antioxidant study of vitamins B (niacin and niacin amide) and C (ascorbic acid) with DPPH through UV spectrophotometric and physicochemical methods. Journal of Molecular Liquids.

[bb0025] Balti R., Mansour M.B., Sayari N., Yacoubi L., Rabaoui L., Brodu N., Massé A. (2017). Development and characterization of bioactive edible films from spider crab (Maja crispata) chitosan incorporated with Spirulina extract. International Journal of Biological Macromolecules.

[bb0030] Choi I., Lee J.Y., Lacroix M., Han J. (2017). Intelligent pH indicator film composed of agar/potato starch and anthocyanin extracts from purple sweet potato. Food Chemistry.

[bb0035] En C.L., Nawawi N.I.M., Ijod G., Hasanah N.N., Abedin N.H.Z., Rahman Q., Ismail-Fitry M.R., Azman E.M. (2025). Potato starch/agar-based intelligent films infused with dried blackcurrant pomace anthocyanins for freshness monitoring of freshwater prawns. International Food Research Journal.

[bb0040] Eze F.N., Jayeoye T.J., Singh S. (2022). Fabrication of intelligent pH-sensing films with antioxidant potential for monitoring shrimp freshness via the fortification of chitosan matrix with broken Riceberry phenolic extract. Food Chemistry.

[bb0045] Ge J., Yu X.Y., Wang S., Chi J.P., Liang J., Sun Y., Gao X.L., Yue P.X. (2019). Nanocomplexes composed of chitosan derivatives and β-Lactoglobulin as a carrier for anthocyanins: Preparation, stability and bioavailability in vitro. Food Research International.

[bb0050] Guo C., Li Y., Zhang H., Zhang Q.Y., Wu X.D., Wang Y., Sun F.D., Shi S., Xia X.F. (2024). A review on improving the sensitivity and color stability of naturally sourced pH-sensitive indicator films. Comprehensive Reviews in Food Science and Food Safety.

[bb0055] Guo L.K., Xu Y.J., Xu Q.H., Jin L.Q. (2024). Nanocellulose-lignin films incorporating AgNPs for potential wound dressing: Green synthesis, antioxidant ability, antibacterial capability and biocompatibility. Cellulose.

[bb0060] Hao R.Y., Pang S.W., Mraz J., Geng Y.Y., Liu Y.Q., Pan J.F. (2024). Anthocyanin modified by chondroitin sulphate and tannic acid improved the quality-indicating properties of gelatin-based intelligent film. Food Chemistry: X.

[bb0065] Hu H., Yong H., Yao X., Yun D., Huang J., Liu J. (2021). Highly efficient synthesis and characterization of starch aldehyde-catechin conjugate with potent antioxidant activity. International Journal of Biological Macromolecules.

[bb0070] Huang H.L., Tsai I.L., Lin C., Hang Y.H., Ho Y.C., Tsai M.L., Mi F.L. (2023). Intelligent films of marine polysaccharides and purple cauliflower extract for food packaging and spoilage monitoring. Carbohydrate Polymers.

[bb0075] Huang X., Du L., Li Z., Xue J., Shi J.Y., Tahir H.E., Zou X.B. (2023). A visual bi-layer indicator based on mulberry anthocyanins with high stability for monitoring Chinese mitten crab freshness. Food Chemistry.

[bb0080] Huang Y.X., Zhou S.Y., Zhao G.H., Ye F.Y. (2021). Destabilisation and stabilisation of anthocyanins in purple-fleshed sweet potatoes: A review. Trends in Food Science & Technology.

[bb0085] Jamróz E., Kopel P. (2020). Polysaccharide and protein films with antimicrobial/antioxidant activity in the food industry: A review. Polymers.

[bb0090] Jiang G., Hou X., Zeng X., Zhang C., Wu H., Shen G., Li S.S., Luo Q.Y., Li M.L., Liu X.Y., Chen A.J., Wang Z.Y., Zhang Z.Q. (2020). Preparation and characterization of indicator films from carboxymethyl-cellulose/starch and purple sweet potato (*Ipomoea batatas* (L.) lam) anthocyanins for monitoring fish freshness. International Journal of Biological Macromolecules.

[bb0095] Jiang H., Zhang W., Pu Y., Chen L., Cao J., Jiang W. (2023). Development and characterization of a novel active and intelligent film based on pectin and betacyanins from peel waste of pitaya (*Hylocereus undatus*). Food Chemistry.

[bb0100] Jin L.Q., Li W.G., Xu Q.H., Sun Q.C. (2015). Amino-functionalized nanocrystalline cellulose as an adsorbent for anionic dyes. Cellulose.

[bb0105] Karnwal A., Rauf A., Jassim A.Y., Selvaraj M., Al-Tawaha A.R.M.S., Kashyap P., Kumar D., Malik T. (2025). Advanced starch-based films for food packaging: Innovations in sustainability and functional properties. Food Chemistry*:* X.

[bb0110] Koosha M., Hamedi S. (2019). Intelligent chitosan/PVA nanocomposite films containing black carrot anthocyanin and bentonite nanoclays with improved mechanical, thermal and antibacterial properties. Progress in Organic Coatings.

[bb0115] Kurek M., Garofulić I.E., Bakić M.T., Ščetar M., Uzelac V.D., Galić K. (2018). Development and evaluation of a novel antioxidant and pH indicator film based on chitosan and food waste sources of antioxidants. Food Hydrocolloids.

[bb0120] Li L.L., Wang W.X., Zheng M.D., Sun J.F., Chen Z.Z., Wang J., Ma Q.Y. (2023). Nanocellulose-enhanced smart film for the accurate monitoring of shrimp freshness via anthocyanin-induced color changes. Carbohydrate Polymers.

[bb0125] Li M., Guo L.K., Mu Y.X., Huang X.D., Jin L.Q., Xu Q.H., Wang Y.L. (2024). Gelatin films reinforced by tannin-nanocellulose microgel with improved mechanical and barrier properties for sustainable active food packaging. Food Hydrocolloids.

[bb0130] Lin H., Wang B., Weng Y. (2020). Development and characterization of sodium caseinate edible films cross-linked with genipin. LWT - Food Science and Technology.

[bb0135] Liu L.M., Wu W.N., Zheng L.M., Yu J.H., Sun P.L., Shao P. (2022). Intelligent packaging films incorporated with anthocyanins-loaded ovalbumin-carboxymethyl cellulose nanocomplexes for food freshness monitoring. Food Chemistry.

[bb0140] Mohammadalinejhad S., Almasi H., Moradi M. (2020). Immobilization of *Echium amoenum* anthocyanins into bacterial cellulose film: A novel colorimetric pH indicator for freshness/spoilage monitoring of shrimp. Food Control.

[bb0145] Moradi M., Tajik H., Almasi H., Forough M., Ezati P. (2019). A novel pH-sensing indicator based on bacterial cellulose nanofibers and black carrot anthocyanins for monitoring fish freshness. Carbohydrate Polymers.

[bb0150] Mosleh Y., de Zeeuw W., Nijemesland M., Bijleveld J.C., van Duin P., Poulis J.A. (2021). The structure-property correlations in dry gelatin adhesive films. Advanced Engineering Materials.

[bb0155] Mu L., Bi J.R., Zhao H.X., Li J.L., Hou H.M., Zhang G.L., Hao H.S., Zhou L. (2025). Intelligent pH-responsive films based on natural blueberry anthocyanins: A non-destructive monitoring system for the freshness of aquatic products with prospective smartphone compatibility. Food Chemistry*: X*.

[bb0160] de Oliveira V.S., Ferreira F.S., Cople M.C.R., Labre T.D., Augusta I.M., Gamallo O.D., Saldanha T. (2018). Use of natural antioxidants in the inhibition of cholesterol oxidation: A review. Comprehensive Reviews in Food Science and Food Safety.

[bb0170] Qin Y., Liu Y.P., Yong H.M., Liu J., Zhang X., Liu J. (2019). Preparation and characterization of active and intelligent packaging films based on cassava starch and anthocyanins from Lycium ruthenicum Murr. International Journal of Biological Macromolecules.

[bb0175] Roy S., Rhim J.W. (2020). Preparation of carbohydrate-based functional composite films incorporated with curcumin. Food Hydrocolloids.

[bb0180] Silva, F. A. G. S., Dourado, F., Gama, M., & Poças, F. (2020). Nanocellulose bio-based composites for food packaging. Nanomaterials, *10*, article 2041. doi:10.3390/nano10102041. PMC7602726 33081126

[bb0185] Sun B., Xu F., Chen D., Liu J. (2025). Quaternary ammonium chitosan-based active packaging films incorporated with dialdehyde guar gum-proanthocyanidins conjugates: Characterization and application in the edible coating of pork. Food Hydrocolloids.

[bb0190] Sun X.H., Zhou T.T., Wei C.H., Lan W.Q., Zhao Y., Pan Y.J., Wu V.C.H. (2018). Antibacterial effect and mechanism of anthocyanin rich Chinese wild blueberry extract on various foodborne pathogens. Food Control.

[bb0195] Swarup R., Jong-Whan R. (2020). Anthocyanin food colorant and its application in pH-responsive color change indicator films. Critical Reviews in Food Science and Nutrition.

[bb0200] Wang S.Y., Han X.Y., Chen Y., Shao Y.K., Song J.Q., Yang C.J., Jian S.Q., Bai R., Ye X., Ding W. (2025). Preparation of sodium alginate/pectin/cellulose nanofibrils films containing black soybean seed coat anthocyanins for monitoring goat meat freshness. Food Control.

[bb0205] Wang Y.X., Liu K., Zhang M., Xu T., Du H.S., Pang B., Si C.L. (2023). Sustainable polysaccharide-based materials for intelligent packaging. Carbohydrate Polymers.

[bb0210] Wen P., Wu J.L., Wu J.H., Wang H., Wu H. (2024). A colorimetric nanofiber film based on ethyl cellulose/gelatin/purple sweet potato anthocyanins for monitoring pork freshness. Foods.

[bb0215] Xu M.Y., Fang D.L., Kimatu B.M., Lyu L., Wu W.L., Cao F.L., Li W.L. (2024). Recent advances in anthocyanin-based films and its application in sustainable intelligent food packaging: A review. Food Control.

[bb0220] Yang S., Ding Q.J., Li Y., Han W.J. (2024). Bacterial cellulose/gelatin-based pH-responsive functional film for food freshness monitoring. International Journal of Biological Macromolecules.

[bb0225] Yao Q.B., Huang F., Lu Y.H., Huang J.M., Ali M., Jia X.Z., Zeng X.A., Huang Y.Y. (2024). Polysaccharide-based food packaging and intelligent packaging applications: A comprehensive review. Trends in Food Science & Technology.

[bb0230] Yong H.M., Hu H., Wang Z., Yun D., Kan J., Liu J. (2022). Structure, stability and antioxidant activity of dialdehyde starch grafted with epicatechin, epicatechin gallate, epigallocatechin and epigallocatechin gallate. Journal of the Science of Food and Agriculture.

[bb0235] Yong H.M., Liu J. (2024). Polysaccharide-catechin conjugates: Synthesis methods, structural characteristics, physicochemical properties, bioactivities and potential applications in food industry. Trends in Food Science & Technology.

[bb0240] Yong H.M., Wang X., Zhang X., Liu Y., Qin Y., Liu J. (2019). Effects of anthocyanin rich purple and black eggplant extracts on the physical, antioxidant and pH-sensitive properties of chitosan film. Food Hydrocolloids.

[bb0245] Yun D.W., Tang C., Chen D., Yong H.M., Liu J. (2026). Comparison of the structural characteristics, pH-sensitivity, antioxidant activity and thermal stability of different dialdehyde polysaccharides grafted with purple sweet potato anthocyanins. Food Chemistry.

[bb0250] Yun D.W., Yong H.M., Xu F.F., Li N., Guan T.Z., Liu J. (2025). Purple sweet potato anthocyanin-g-dialdehyde locust bean gum: An innovative polymeric colorant with good stability and application potential in food intelligent packaging. Food Hydrocolloids.

[bb0255] Zhang J., Zou X., Zhai X., Huang X., Jiang C., Holmes M. (2019). Preparation of an intelligent pH film based on biodegradable polymers and roselle anthocyanins for monitoring pork freshness. Food Chemistry.

[bb0260] Zhang J.R., Wang Y.X., Xu T., Li W.R., Dong Q., Zhang M., Qi J.J., Zhang H., Wang X., Liu W. (2025). Strategies, mechanism, and prospects for wood biomass-based intelligent food packaging materials. Trends in Food Science & Technology.

[bb0265] Zhang K.L., Huang T.S., Yan H., Hu X.Z., Ren T. (2020). Novel pH-sensitive films based on starch/polyvinyl alcohol and food anthocyanins as a visual indicator of shrimp deterioration. International Journal of Biological Macromolecules.

[bb0270] Zhang R., Zhang Q., Oliveira H., Mateus N., Ye S.X., Jiang S.J., He J.R., Wu M.C. (2022). Preparation of nanoliposomes loaded with anthocyanins from grape skin extracts: Stability, gastric absorption and antiproliferative properties. Food & Function.

[bb0275] Zhang X.Y., Zou W.J., Xia M.Q., Zeng Q., Cai Z.X. (2022). Intelligent colorimetric film incorporated with anthocyanins-loaded ovalbumin-propylene glycol alginate nanocomplexes as a stable pH indicator of monitoring pork freshness. Food Chemistry.

[bb0280] Zhang Y., Tang Q., Huang K., Xu Z., Feng S., Li H., Zou Z. (2023). Developing strong and tough cellulose acetate/ZIF67 intelligent active films for shrimp freshness monitoring. Carbohydrate Polymers.

