# Supplementary material for: Covalent immobilization of anthocyanins onto cellulose nanofibrils for intelligent food packaging films
Source: Food Chem X. 2026 Jul 6;37:104175. doi: 10.1016/j.fochx.2026.104175 (PMC13356651; doi:10.1016/j.fochx.2026.104175)
Supplement: Supplementary file 1 — Supplementary material [file mmc1.docx]

**Supplementary Materials for**

**Covalent immobilization of anthocyanins onto cellulose nanofibrils for intelligent food packaging films**

Jiawei Zheng, Yang Zhang, Xidong Li, Qinghua Xu*, Liqiang Jin*

State Key Laboratory of Green Papermaking and Resource Recycling, Qilu University of Technology (Shandong Academy of Sciences), Jinan250353, China.

*Corresponding author: Qinghua Xu, [xqh@qlu.edu.cn](mailto:xqh@qlu.edu.cn)

Liqiang Jin, jlq@qlu.edu.cn


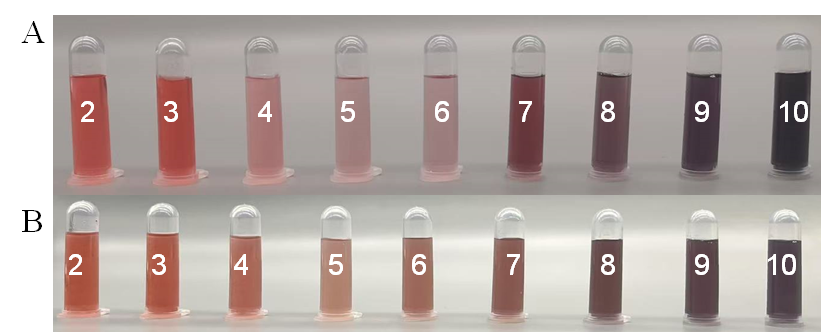


Fig.S1 Color change of A, ACNs and B, ADNF5 at a pH range of 2–10


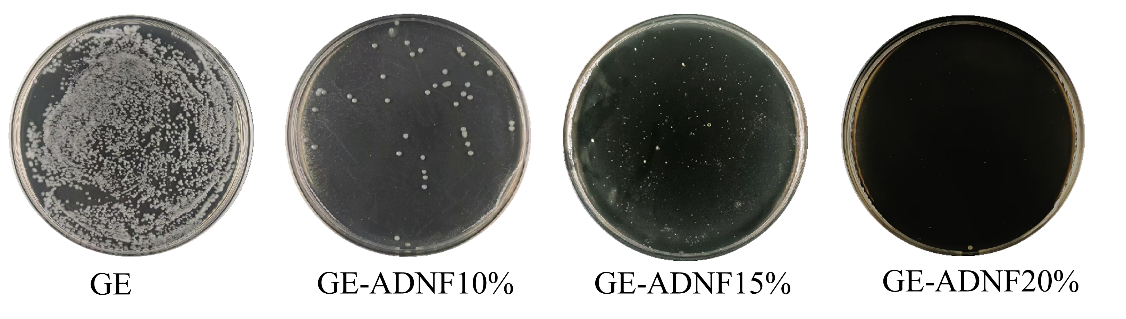


Fig. S2 The colony images of S. aureus cultured on GE and GE-ADNF films

The antibacterial activities of the GE and GE-ADNF films against S. aureus were evaluated using the plate count method according to the literature (Sun et al., 2018).

Sun, X.H., Zhou T.T., Wei C.H., Lan W.Q., Zhao, Y., Pan, Y.J., & Wu, V.C.H. (2018). Antibacterial effect and mechanism of anthocyanin rich Chinese wild blueberry extract on various foodborne pathogens. Food Control, 94, 155-161. https://doi.org/10.1016/j.foodcont.2018.07.012





Fig.S3 Water swelling ratio of GE-ADNF composite films


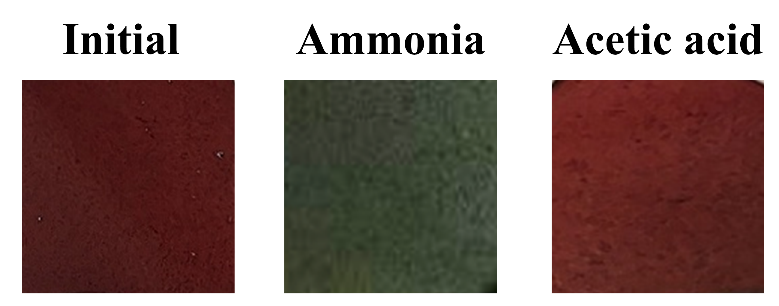


Fig.S4 Color reversibility of GE-ADNF20% film

The color reversibility of GE-ADNF20% film was evaluated according to the previous literature (Huang et al., 2023). The film (30 mm × 40 mm) was exposed to a 25–28% ammonia solution and acetic acid (20 mL, 2 mol/L) solution repeatedly and alternatively for 3 min each. The images of the tested samples in the reversible pH-sensing measures were photographed.

Huang, X., Du, L., Li, Z., Xue J., Shi J.Y., Tahir H. E., Zhai X.D., Zhang J.J., Zhang N., Sun W., Zou X.B. (2023). A visual bi-layer indicator based on mulberry anthocyanins with high stability for monitoring Chinese mitten crab freshness. Food Chemistry, 411, 135497.

<https://doi.org/10.1016/j.foodchem.2023.135497>

**Table S1** Comparison of dry tensile strength (d-TS), wet tensile strength (w-TS), water contact angle (WCA), NH_3_ response time, TVB-N correlation in this work with some other reported intelligent films

| Intelligent films | d-TS  (MPa) | w-TS  (MPa) | WCA  (°) | NH_3_ response time (min) | TVB-N correlation  (R^2^) | Reference |
| --- | --- | --- | --- | --- | --- | --- |
| Starch/PVA/OVA-CMC-ACN | 28.87 | N/A | 87.8 | N/A | N/A | Liu et al., 2022 |
| GE‑purple cabbage anthocyanin (PCA)‑chondroitin sulphate (CS) | 17.97 | N/A | 64.8 | N/A | 0.90487 | Hao, Pang, Mraz, Geng, Liu & Pan, 2024 |
| (BC)/gelatin (GE)/fluorescein isothiocyanate (FITC)/red cabbage (PCA) | 30 | N/A | 82.73 | 3 | N/A | Yang, Ding, Li & Han, 2024 |
| (BC)/black carrot anthocyanins  (CA) | N/A | N/A | N/A | N/A | 0.97 | Moradi, Tajik, Almasi, Forough, & Ezati, 2019 |
| PVA/chitosan/  roselle anthocyanins | 98.28 | N/A | N/A | 24 | N/A | Zhang, Zou, Zhai, Huang, Jiang, & Holmes, 2019 |
| GE-ADNF15% | 104.77 | 2.60 | 110 | 3 | 0.9892 | This work |

**References:**

Hao R.Y., Pang S.W, Mraz J., Geng Y.Y., Liu Y.Q., Pan J.F. (2024). Anthocyanin modified by chondroitin sulphate and tannic acid improved the quality-indicating properties of gelatin-based intelligent film. *Food Chemistry: X, 24*, Article101779. <https://doi.org/10.1016/j.fochx.2024.101779>

Liu, L.M., Wu, W.N., Zheng, L.M., Yu, J.H., Sun, P.L., & Shao, P. (2022). Intelligent packaging films incorporated with anthocyanins-loaded ovalbumin-carboxymethyl cellulose nanocomplexes for food freshness monitoring. *Food Chemistry, 387*, Article 132908. <https://doi.org/10.1016/j.foodchem.2022.132908>

Moradi, M., Tajik, H., Almasi, H., Forough, M., & Ezati, P. (2019). A novel pH-sensing indicator based on bacterial cellulose nanofibers and black carrot anthocyanins for monitoring fish freshness. *Carbohydrate Polymers, 222*, Article 115030. <https://doi.org/10.1016/j.carbpol.2019.115030>

Yang S., Ding Q.J., Li Y., Han W.J. (2024). Bacterial cellulose/gelatin-based pH-responsive functional film for food freshness monitoring. *International Journal of Biological Macromolecules, 259*, 129203. <https://doi.org/10.1016/j.ijbiomac.2024.129203>

Zhang, J., Zou, X., Zhai, X., Huang, X., Jiang, C., & Holmes, M. (2019). Preparation of an intelligent pH film based on biodegradable polymers and roselle anthocyanins for monitoring pork freshness. *Food Chemistry, 272,* 306–312. <https://doi.org/10.1016/j.foodchem.2018.08.041>

Table S2 pH responsiveness of the composite films

| Film | color | pH | | | | | | | | |
| --- | --- | --- | --- | --- | --- | --- | --- | --- | --- | --- |
|  |  | 2 | 3 | 4 | 5 | 6 | 7 | 8 | 9 | 10 |
| GE-  ADNF5% | Appearance | 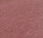 | 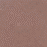 | 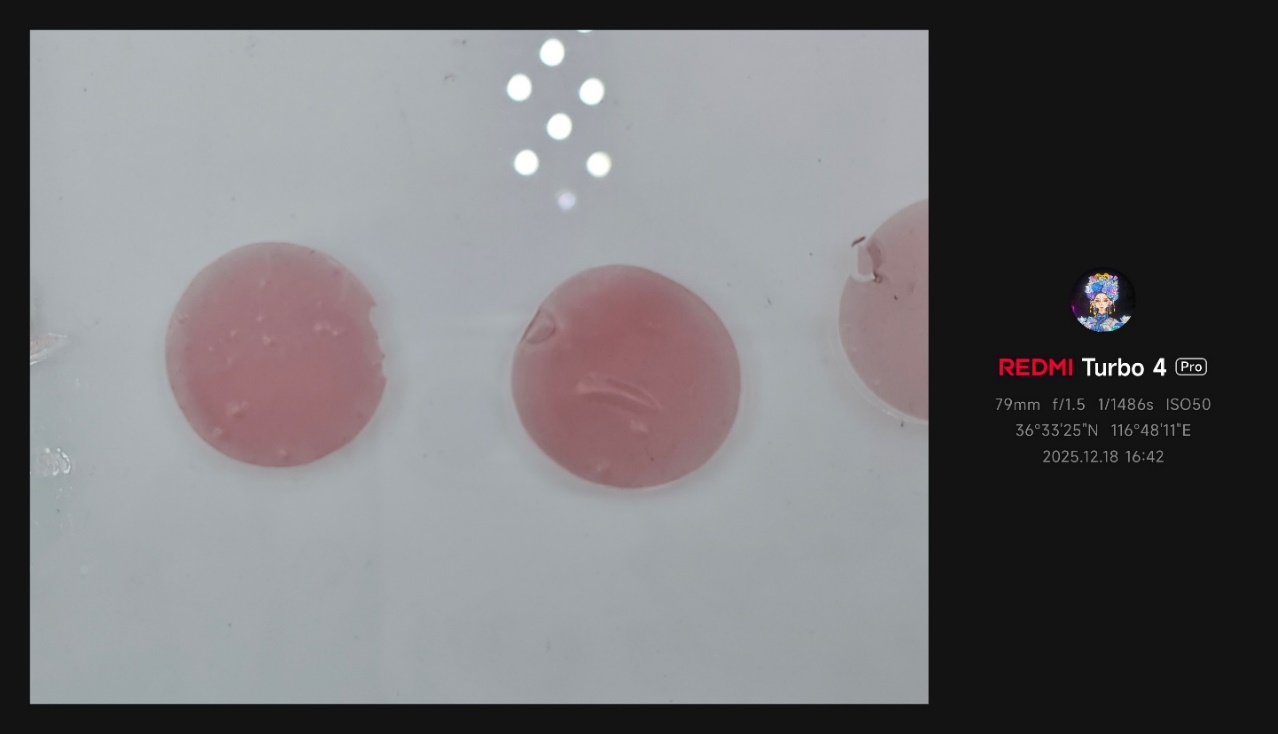 | 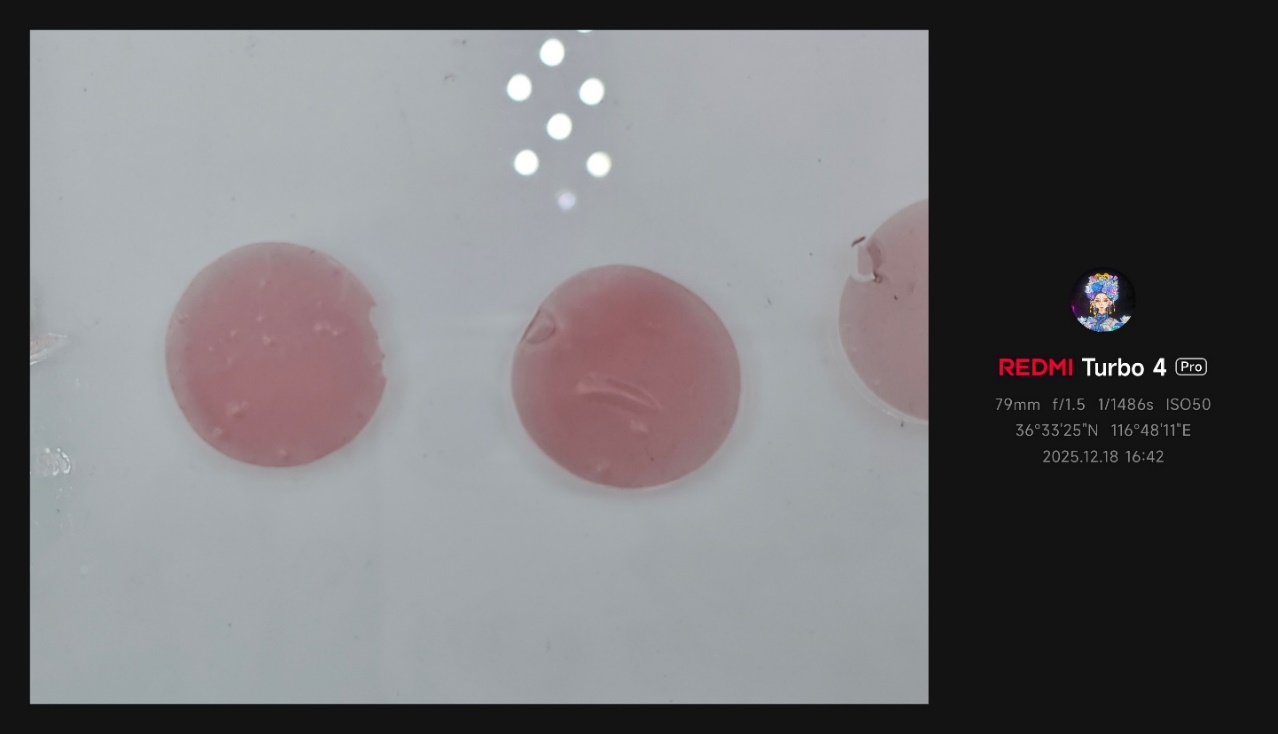 | 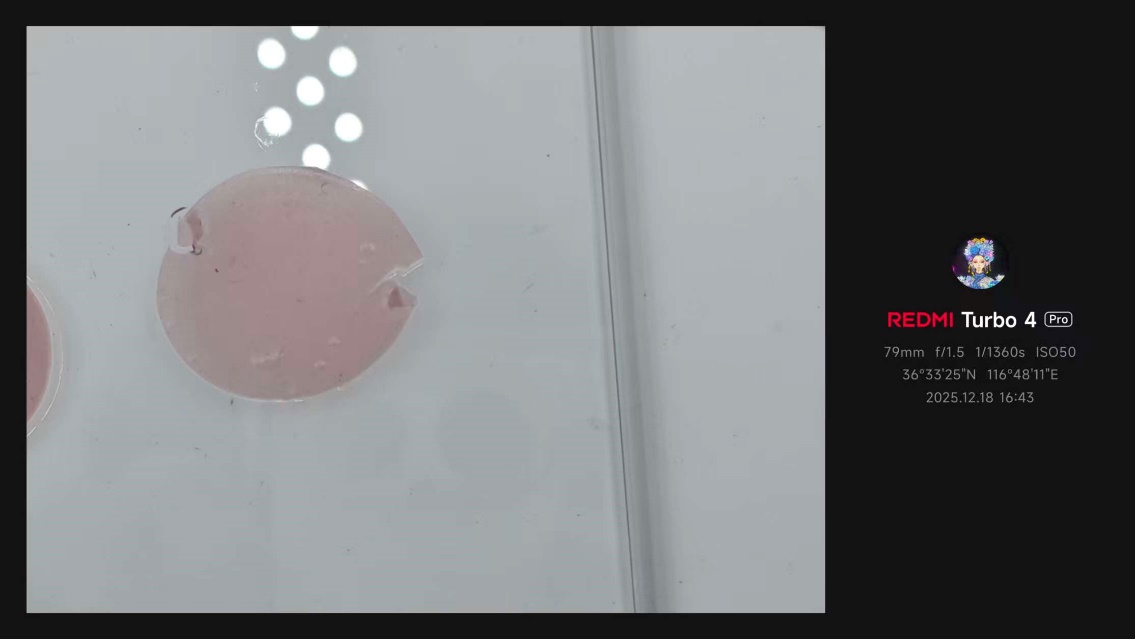 | 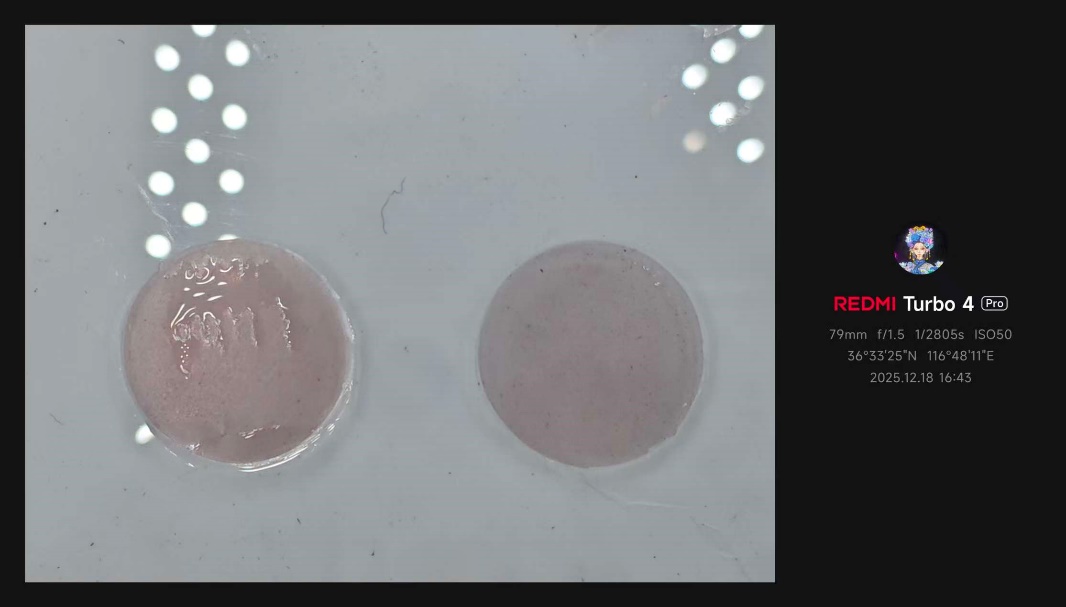 | 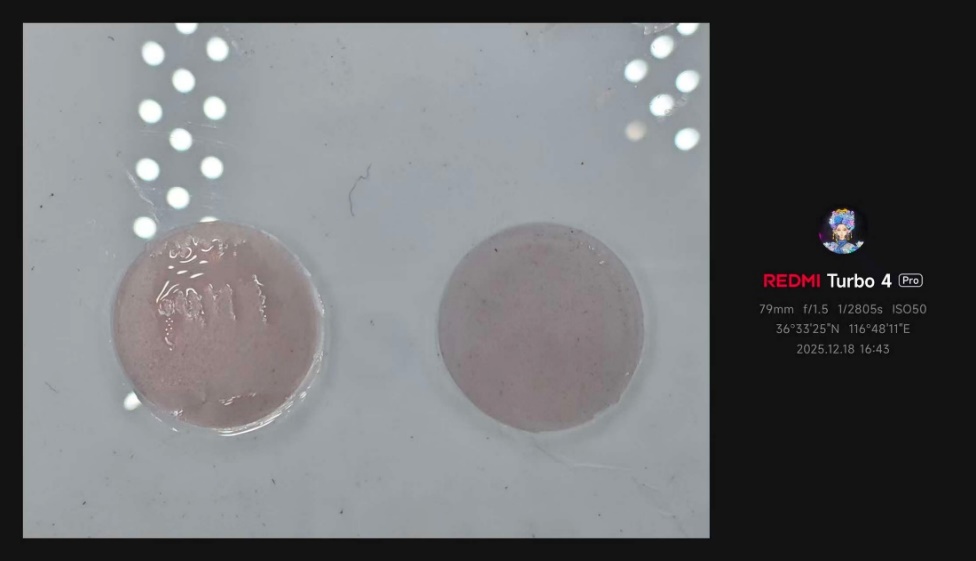 | 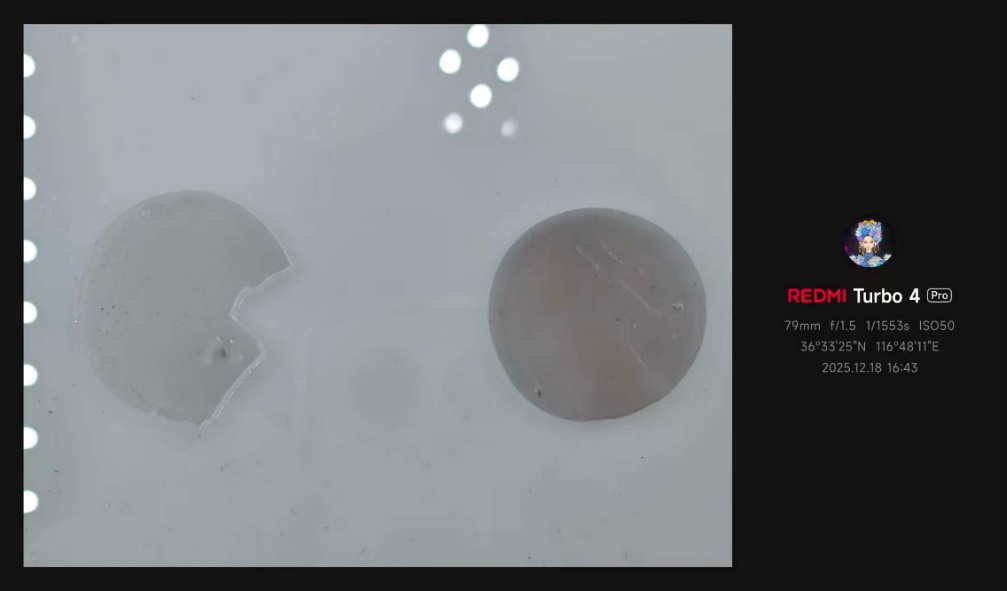 | 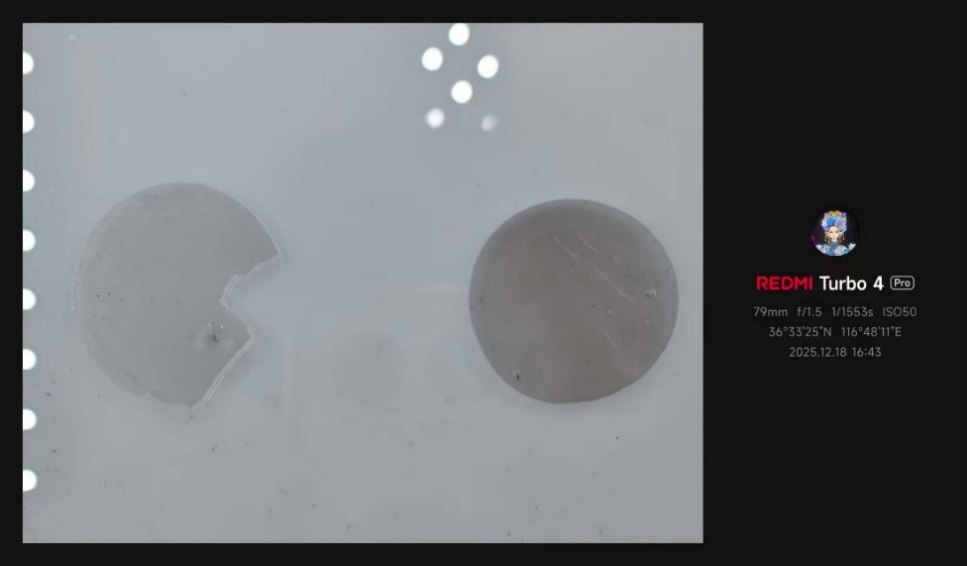 |
|  | L* | 61.9 | 72.6 | 63.3 | 57.1 | 65.3 | 64.7 | 67.8 | 77.7 | 58.6 |
|  | a* | 17.2 | 7.9 | 10 | 11.2 | 7.5 | 6 | 3.6 | 2.5 | 0.9 |
|  | b* | 4.2 | 3.6 | 0.9 | -0.4 | -3.7 | -4.4 | -6.4 | -7.5 | -2.5 |
|  | ΔE | 13.64 | 11.96 | 8.15 | 7.25 | 8.69 | 10.73 | 11.91 | 14.7 | 14.23 |
| GE-  ADNF10% | Appearance | 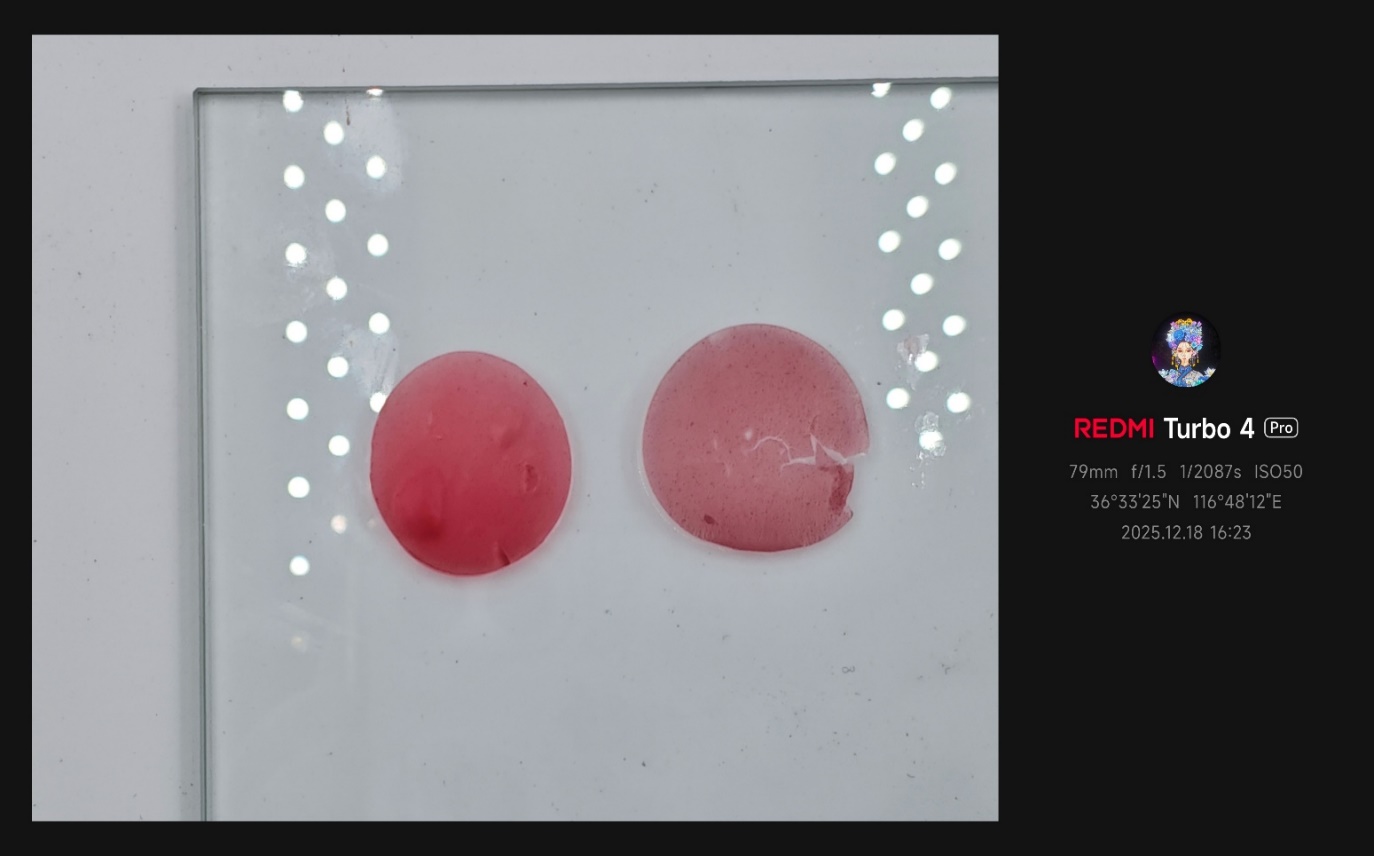 | 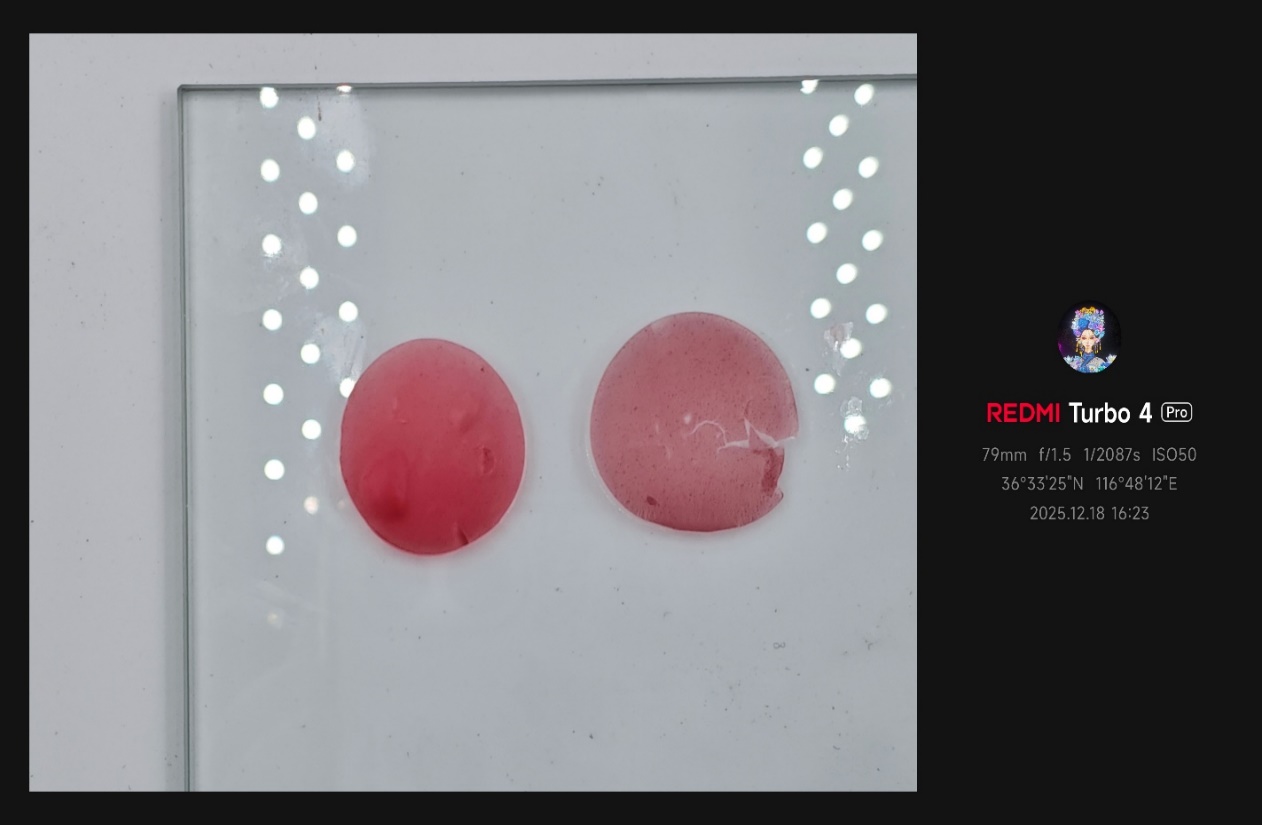 | 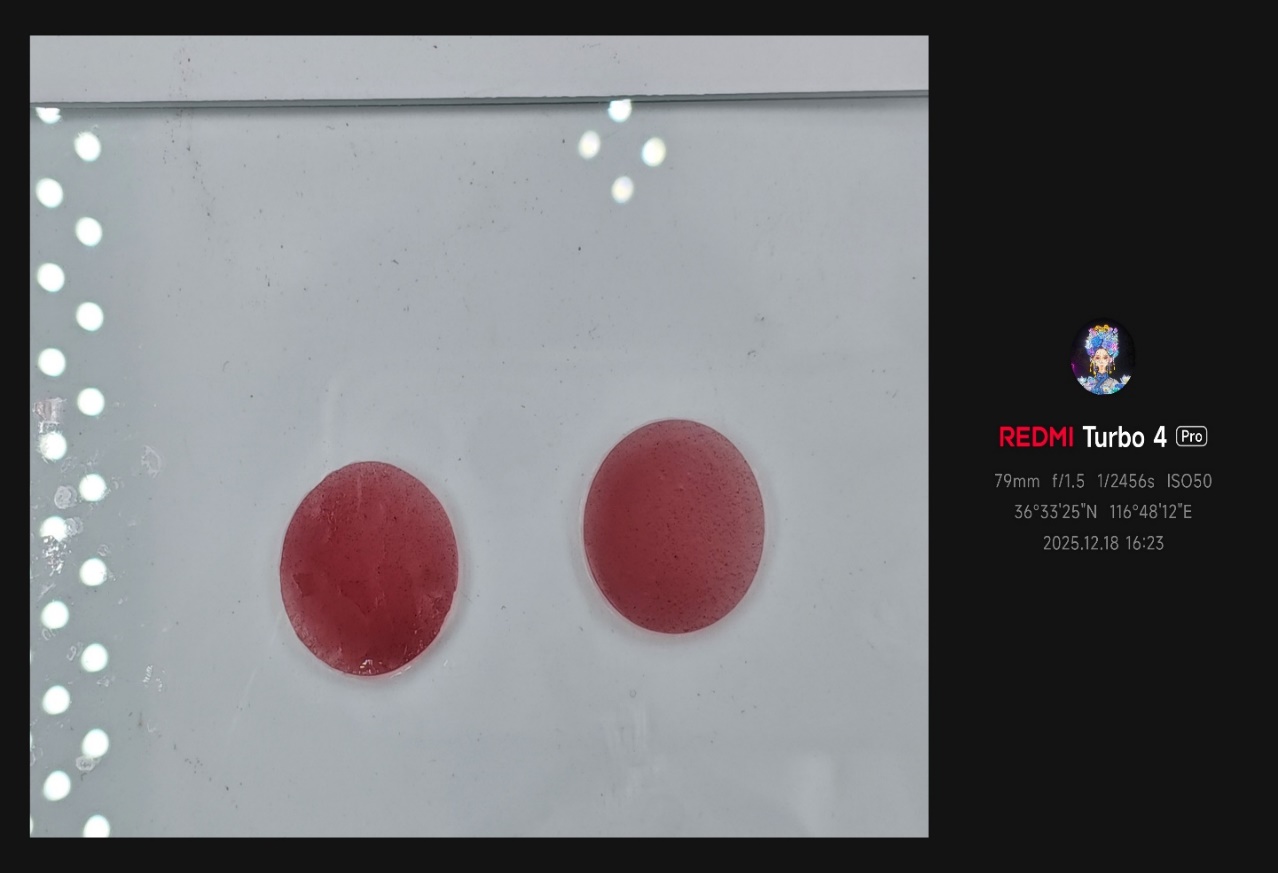 | 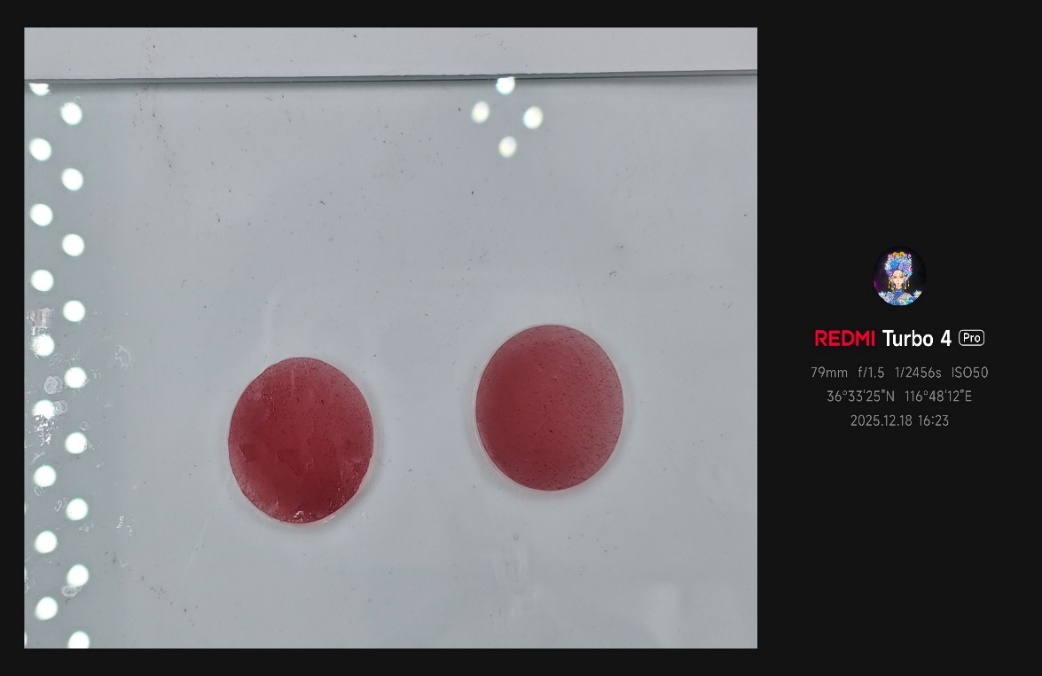 | 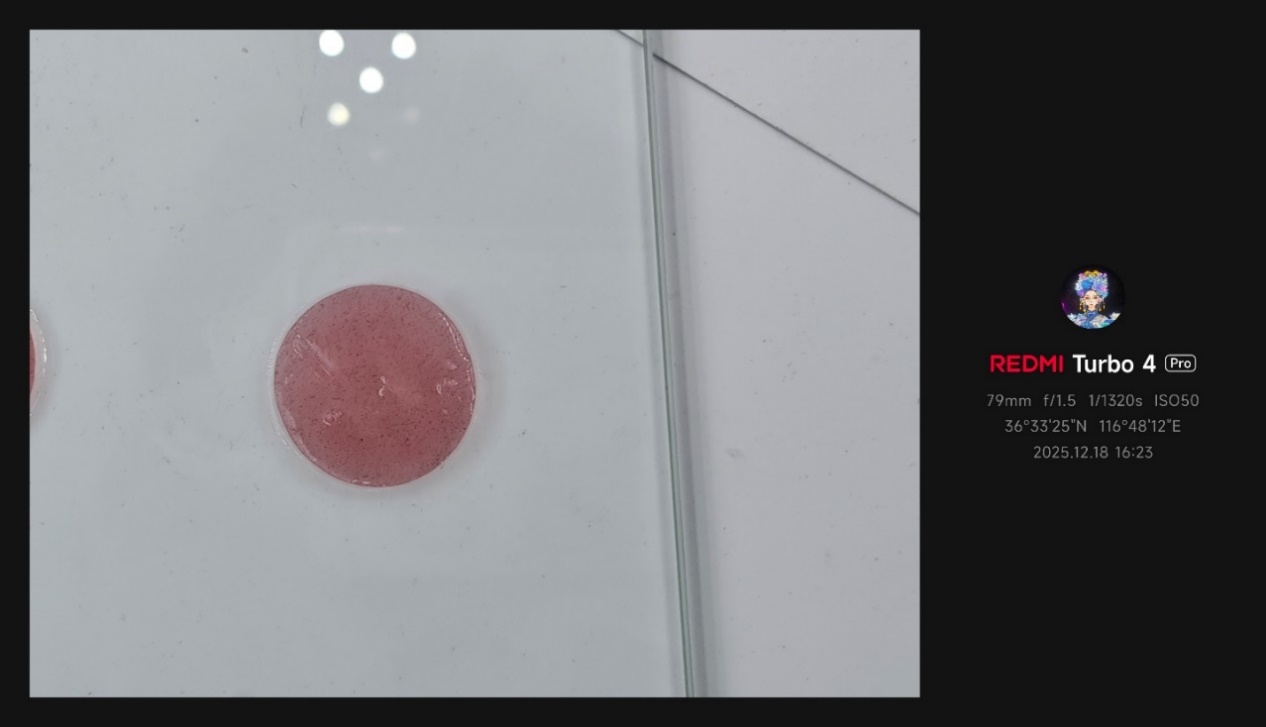 | 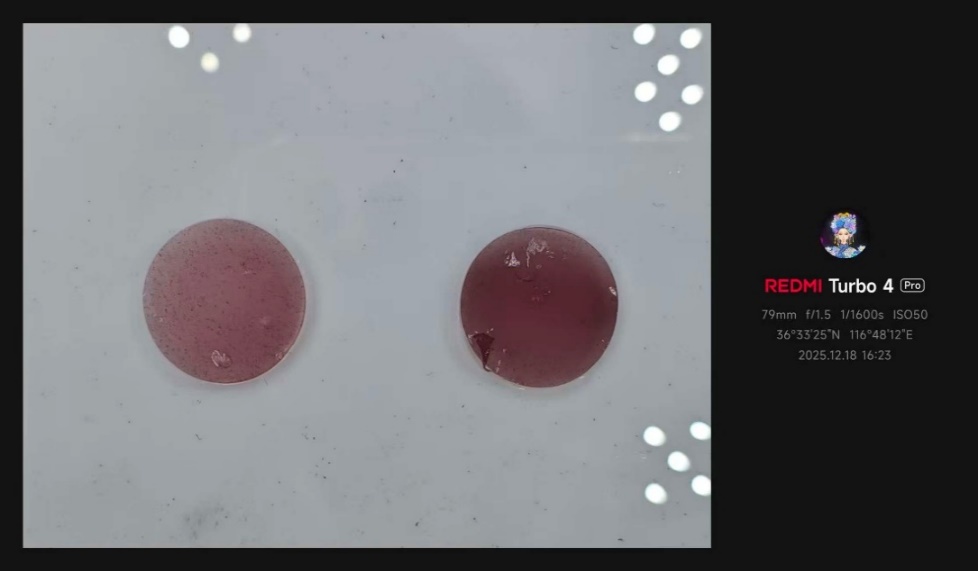 | 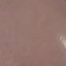 | 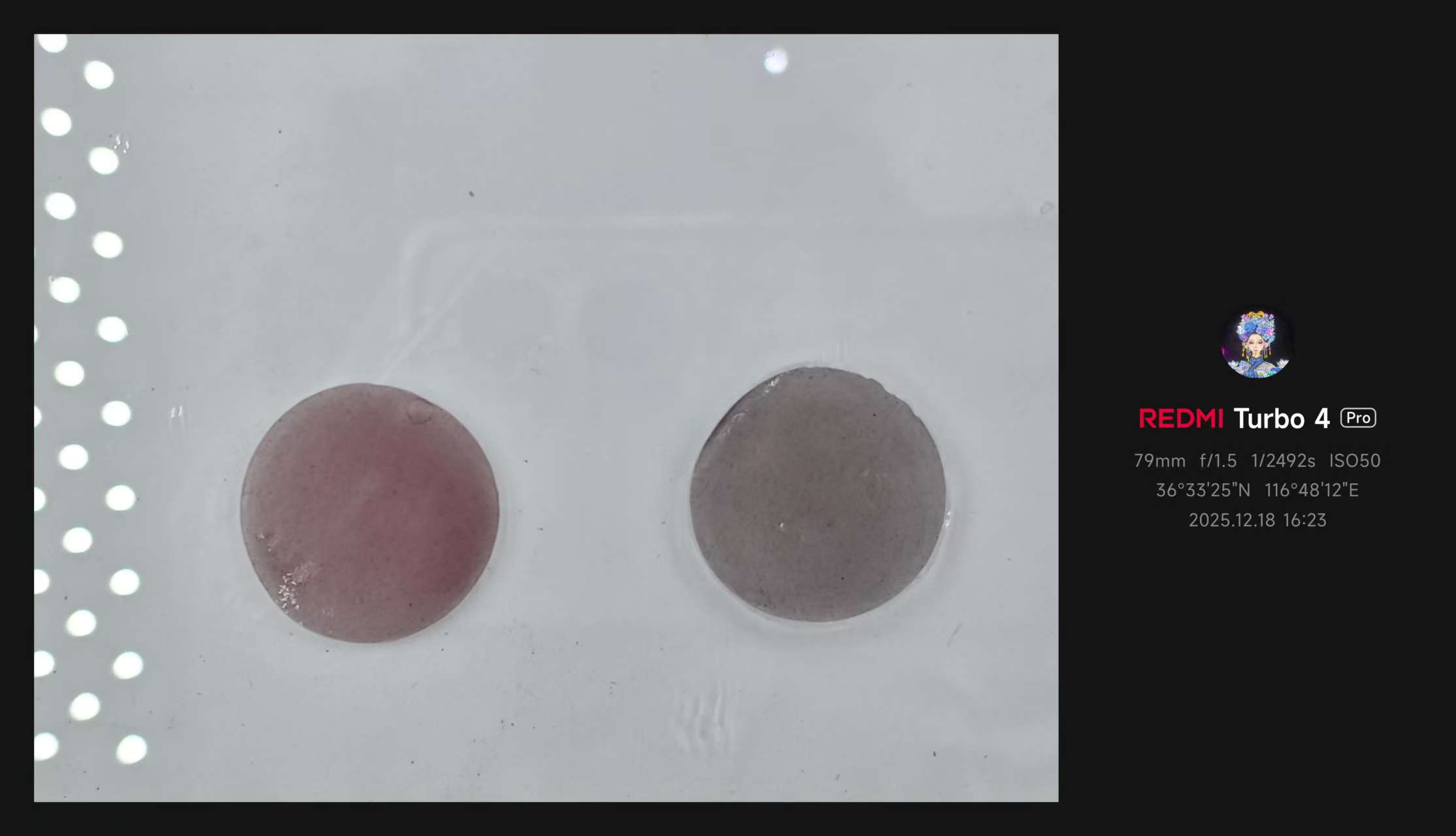 | 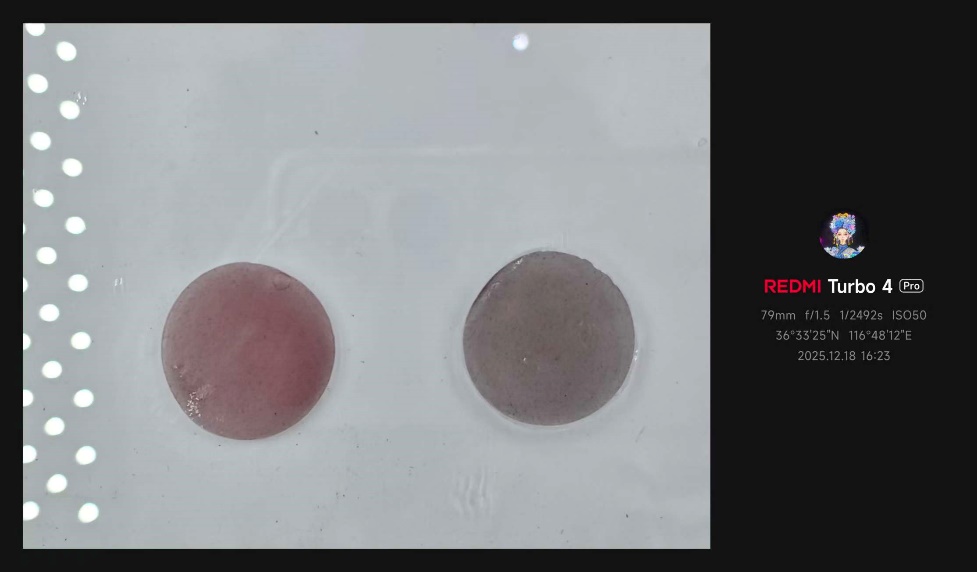 |
|  | L* | 44.1 | 52.7 | 40 | 43.9 | 50.5 | 51.4 | 32.2 | 42.3 | 47 |
|  | a* | 29.1 | 16.8 | 21.2 | 18.3 | 12.6 | 10.4 | 11 | 6.8 | 1.2 |
|  | b* | 3.3 | -0.2 | 5.3 | 4.3 | 1.1 | -0.3 | 3.4 | 0.6 | -0.9 |
|  | ΔE | 10.7 | 8.35 | 9.22 | 3.36 | 6.82 | 10.93 | 16.91 | 20.25 | 23.19 |
| GE-  ADNF15% | Appearance | 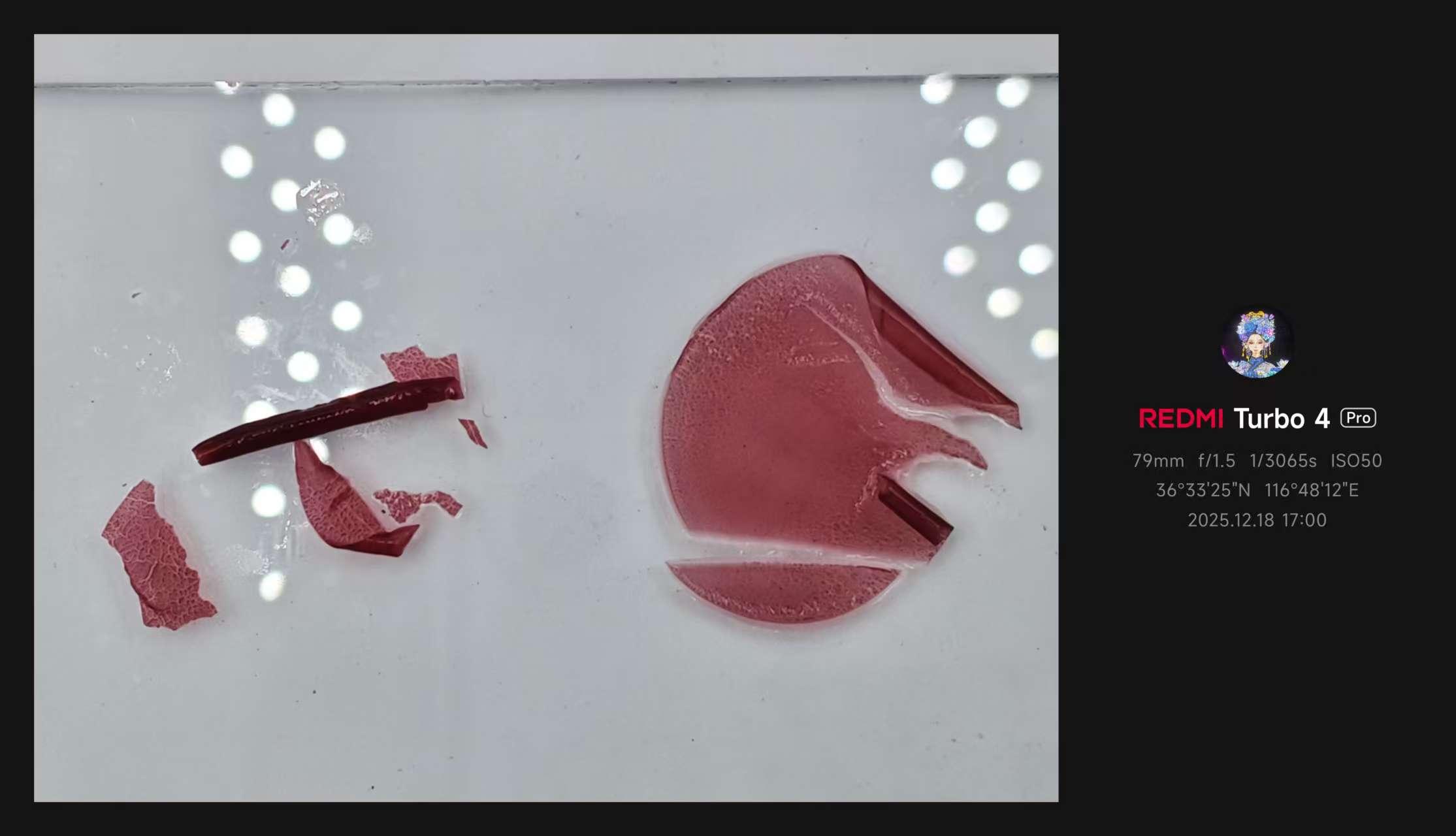 | 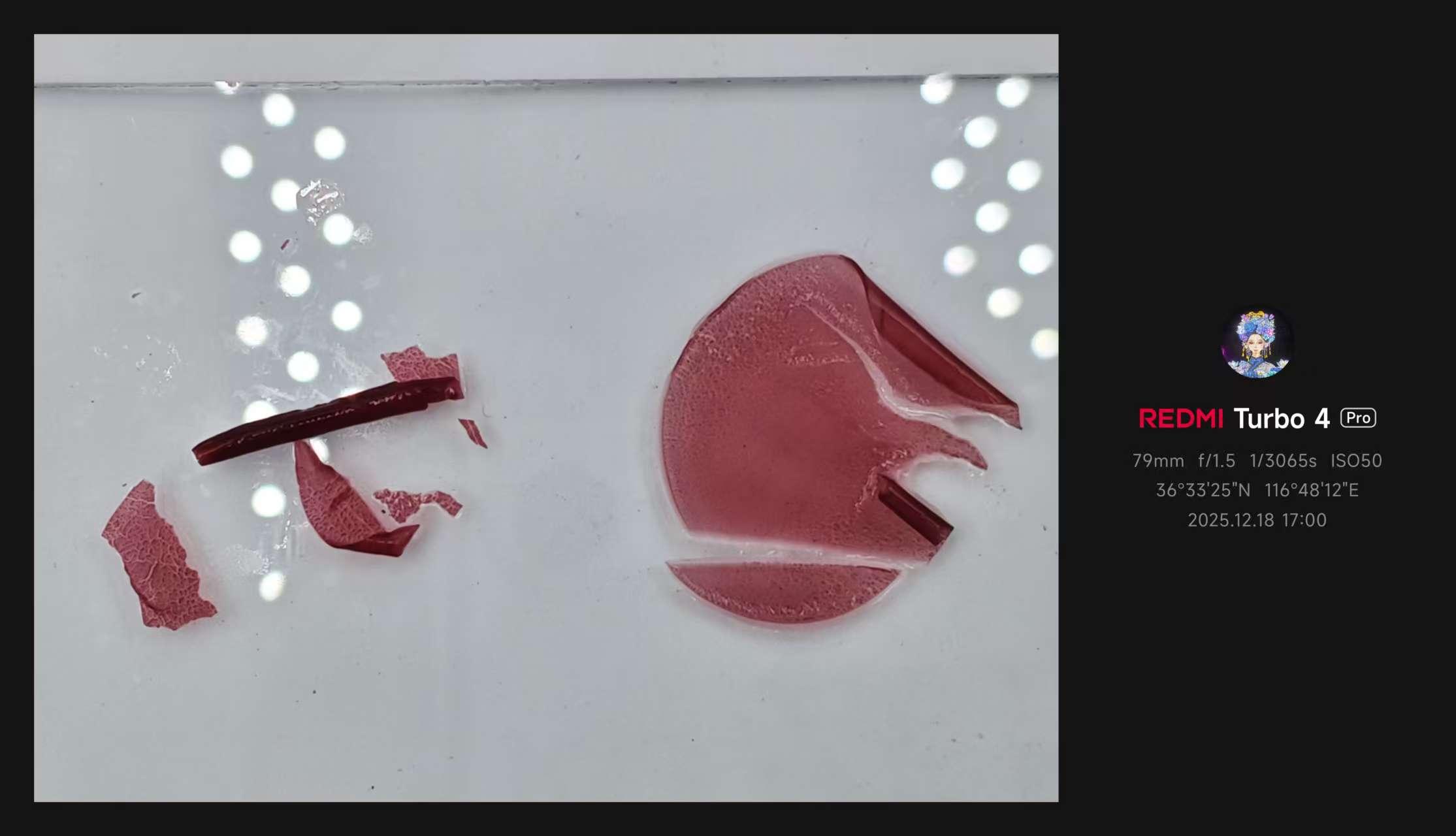 | 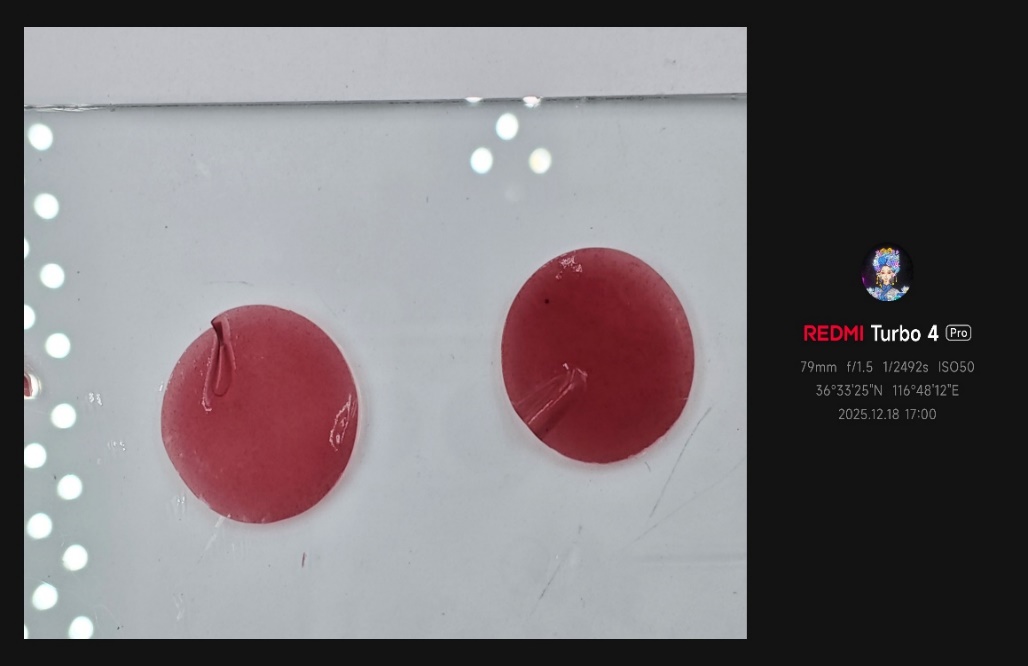 | 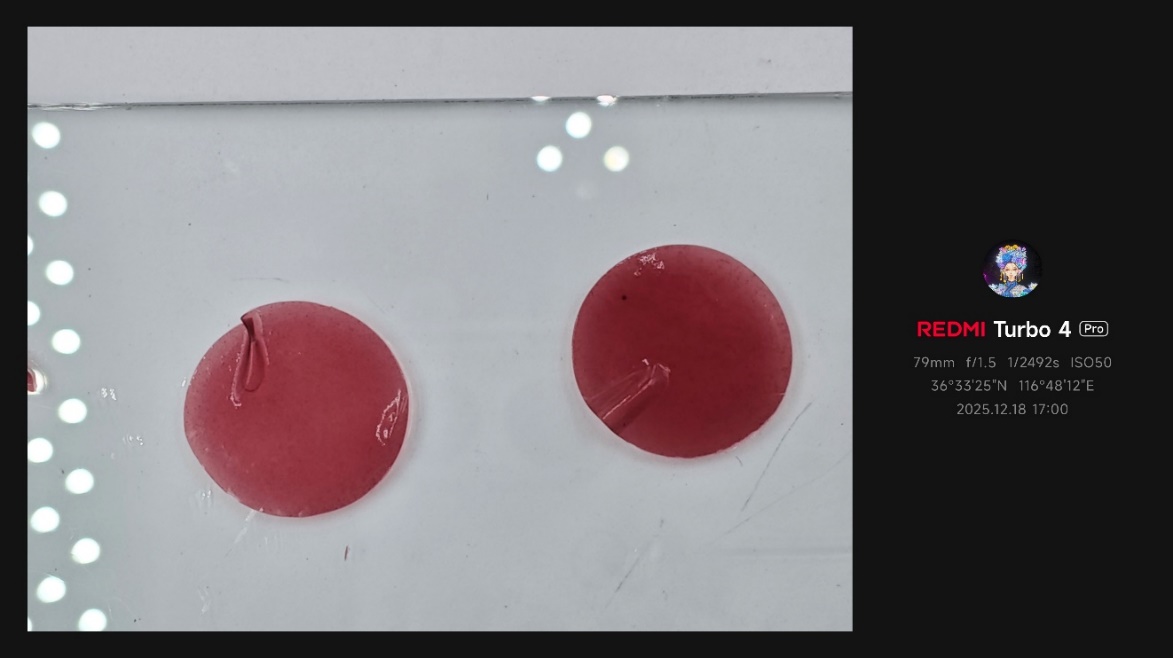 | 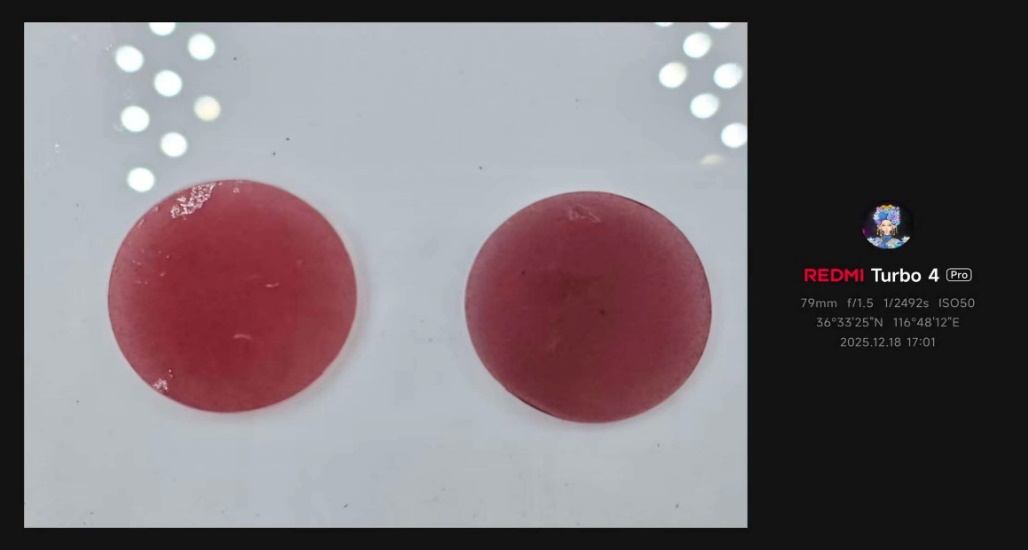 | 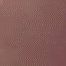 | 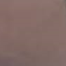 | 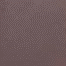 | 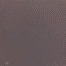 |
|  | L* | 44.1 | 39.2 | 38.2 | 27.1 | 29.4 | 26 | 28.5 | 51.9 | 49.1 |
|  | a* | 27 | 26.8 | 23.6 | 25.8 | 21.6 | 18.2 | 12.9 | 7.4 | 2.5 |
|  | b* | 4.8 | 5 | 6.2 | 8.8 | 7.9 | 6.2 | 3.9 | -2.7 | -0.3 |
|  | ΔE | 15.96 | 13.18 | 1.08 | 4.47 | 8.5 | 9.9 | 16.49 | 22.02 | 23.47 |
| GE-  ADNF20% | Appearance | 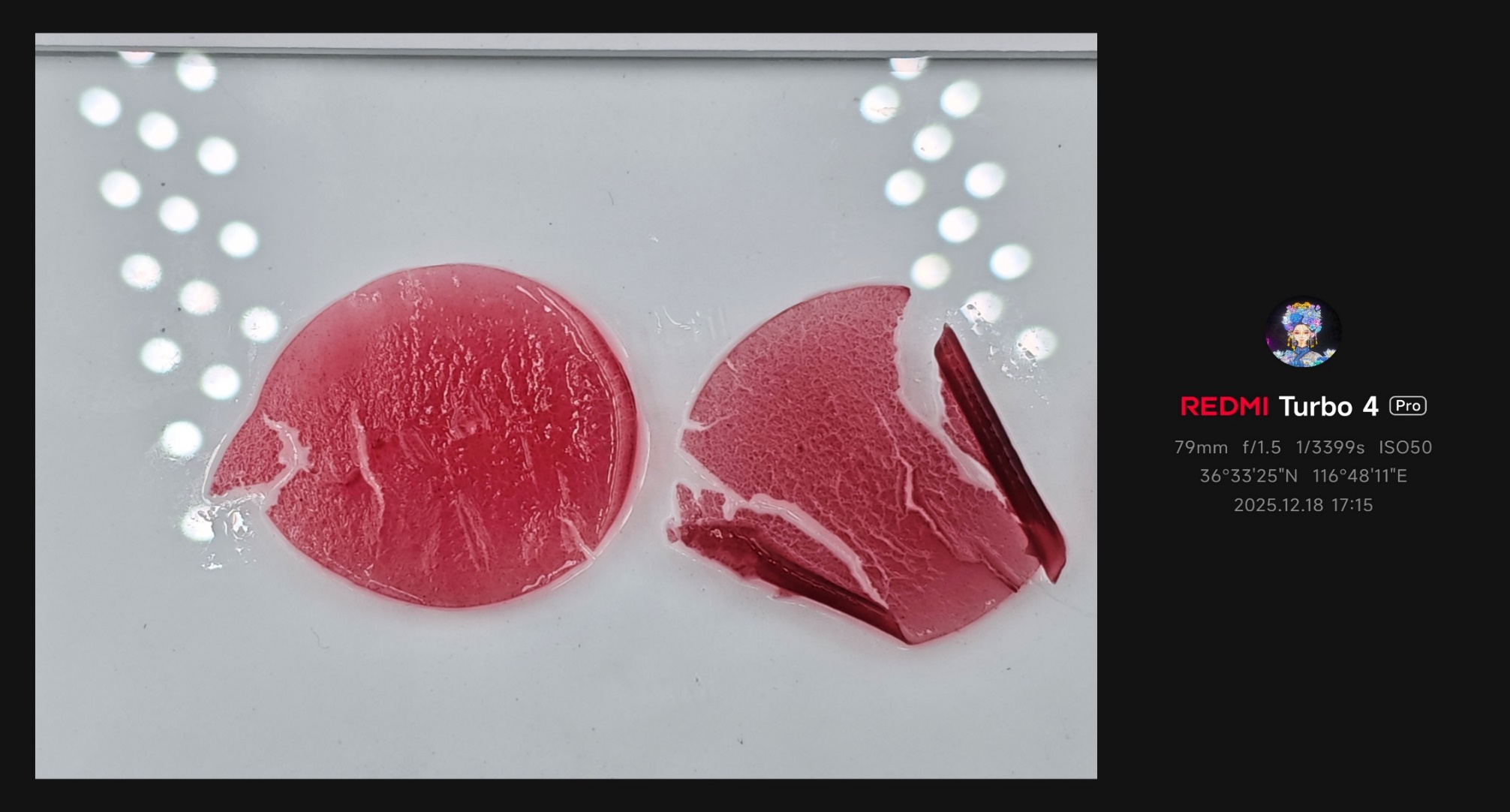 | 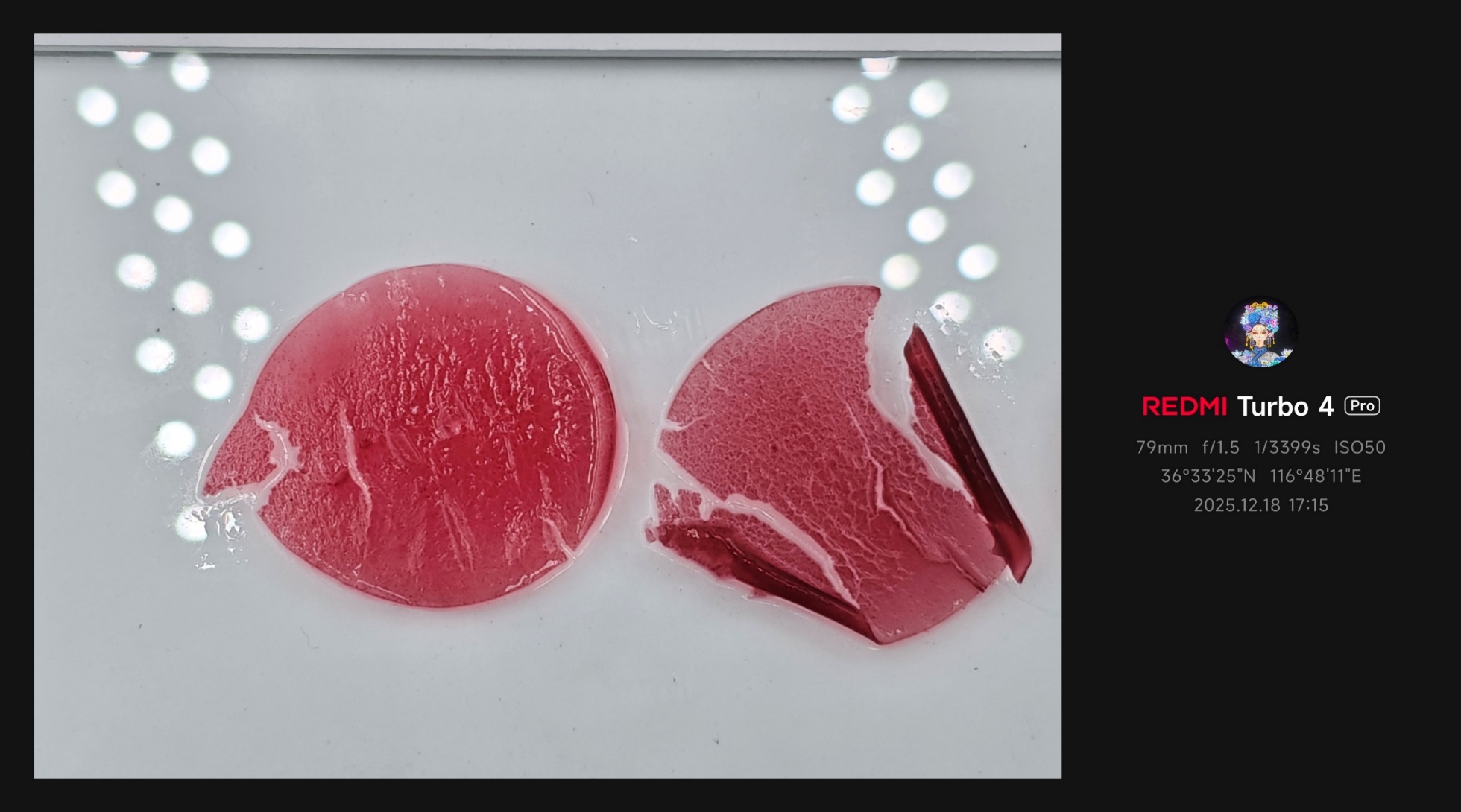 | 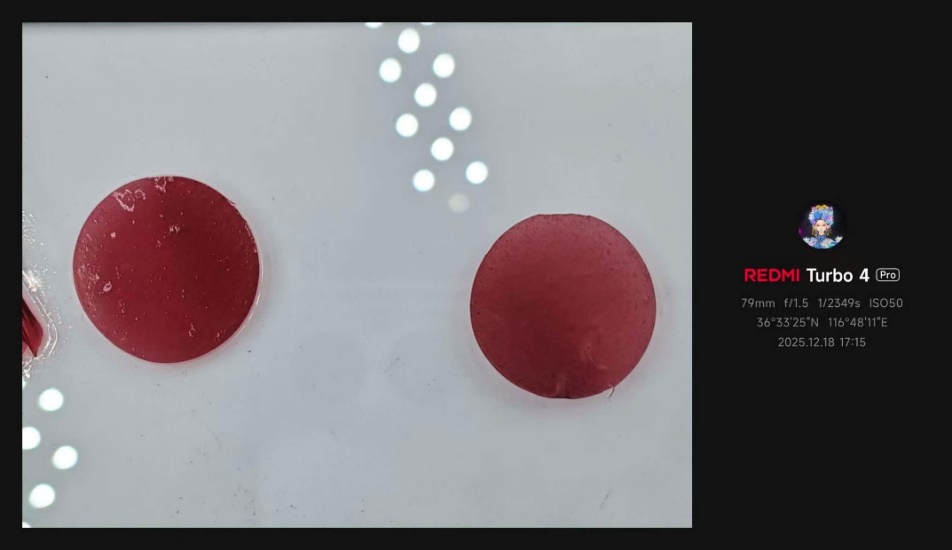 | 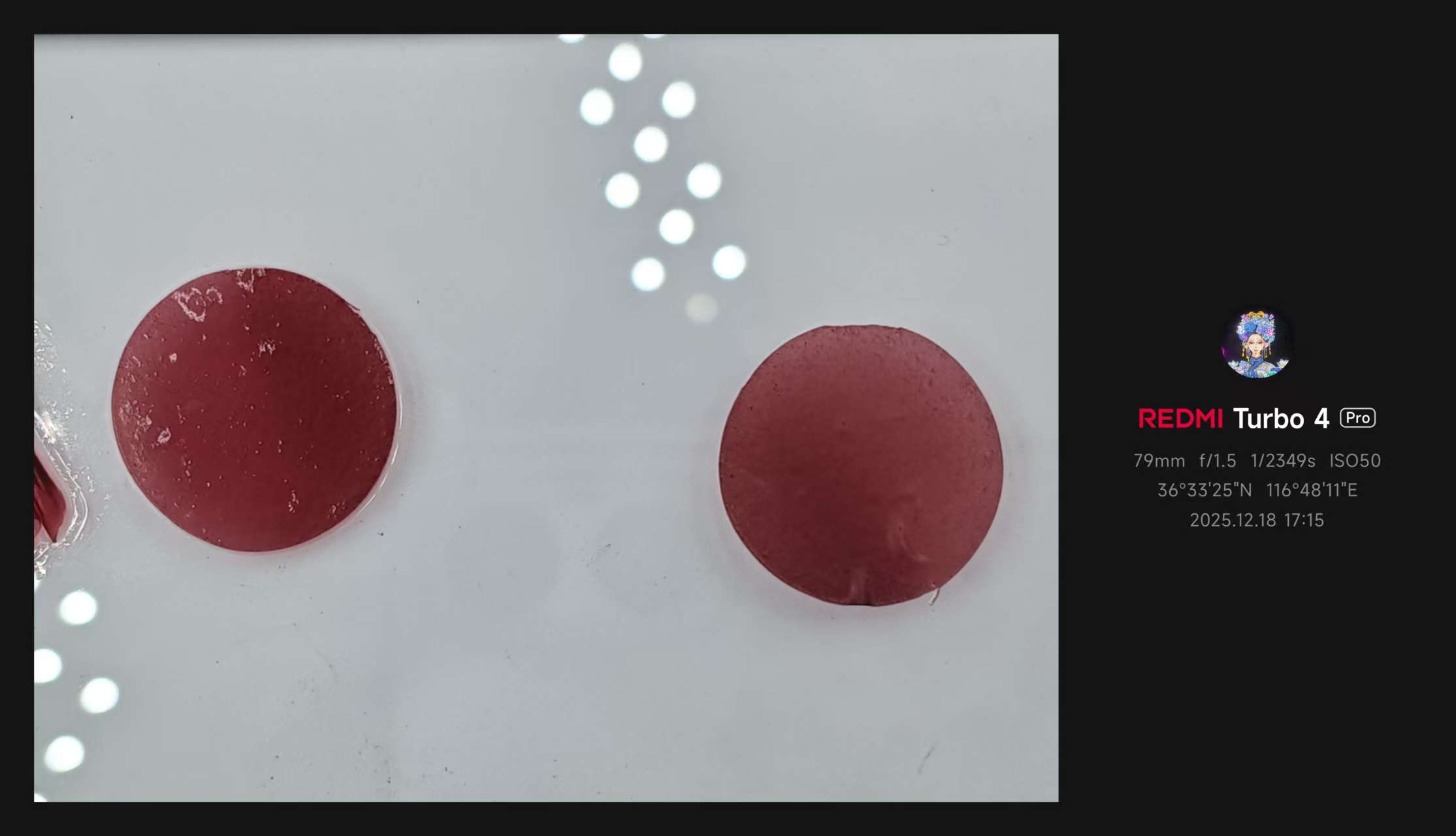 | 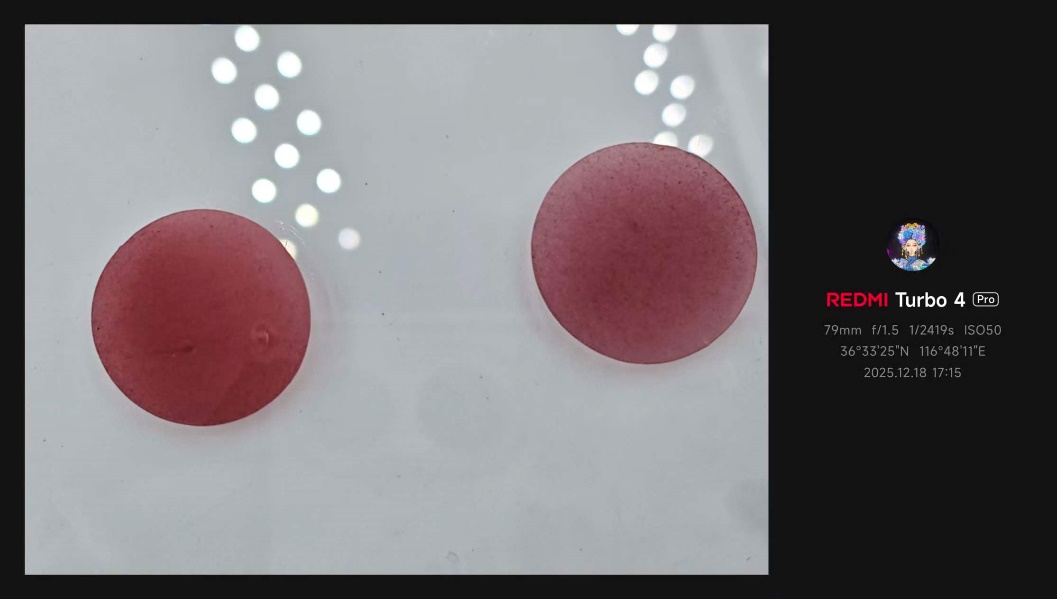 | 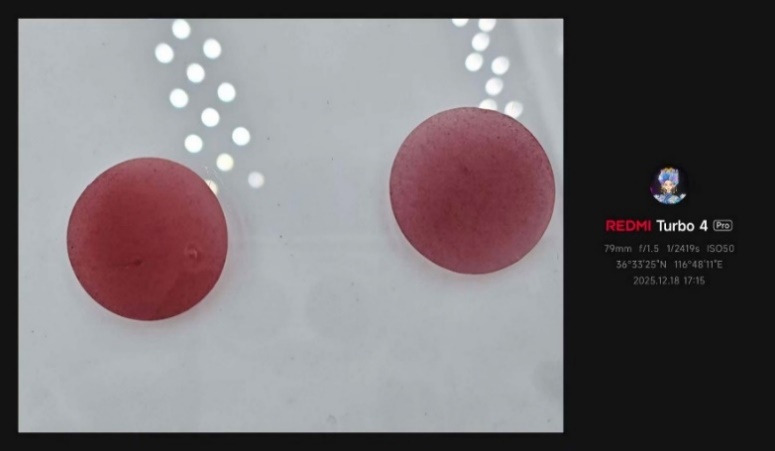 | 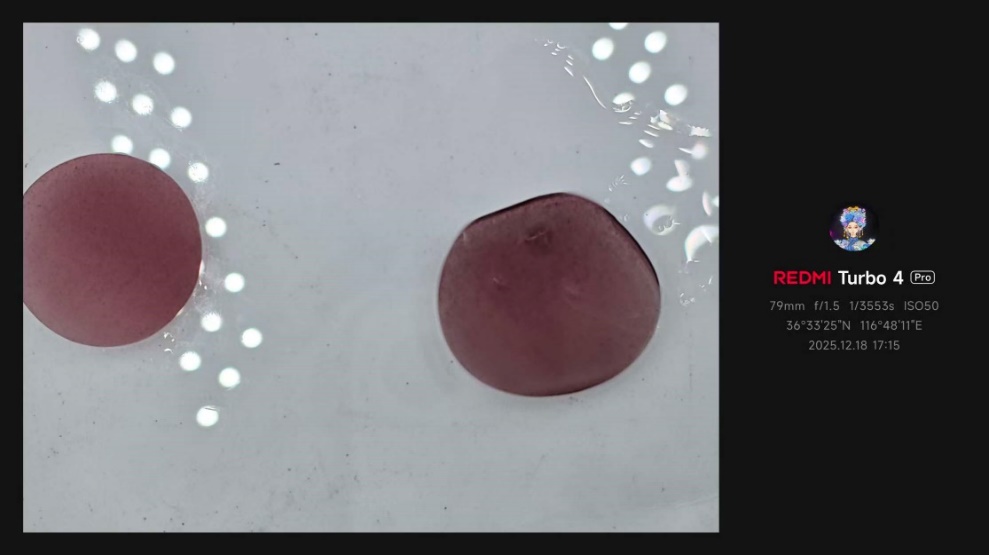 | 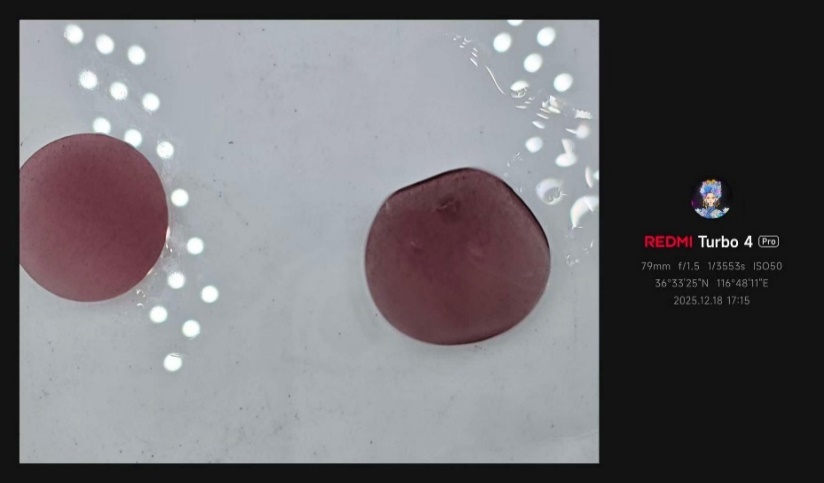 | 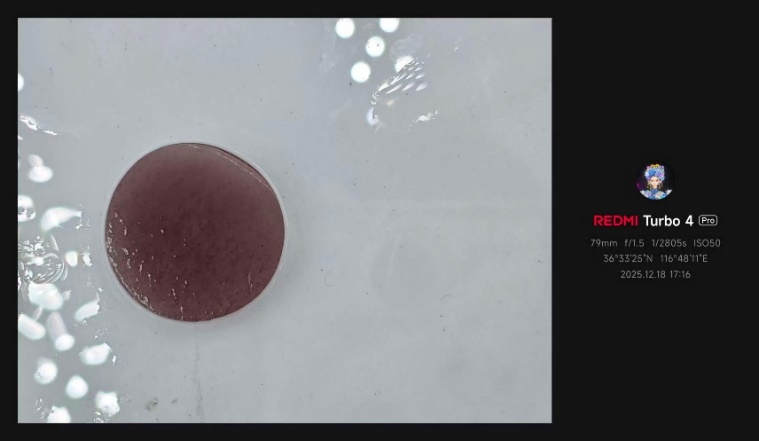 |
|  | L* | 33.7 | 38.2 | 16.1 | 25.3 | 30 | 27.4 | 25.4 | 28 | 24.5 |
|  | a* | 36 | 24.6 | 23.6 | 22.3 | 22.1 | 17.8 | 13.3 | 8.1 | 5.6 |
|  | b* | 9.8 | 3.4 | 7.5 | 7.4 | 7.2 | 4.9 | 3.9 | 1.9 | 4 |
|  | ΔE | 9.93 | 14.53 | 9.68 | 6.68 | 11.05 | 10.34 | 15.76 | 23.47 | 26.24 |
| GE-  ADNF25% | Appearance | 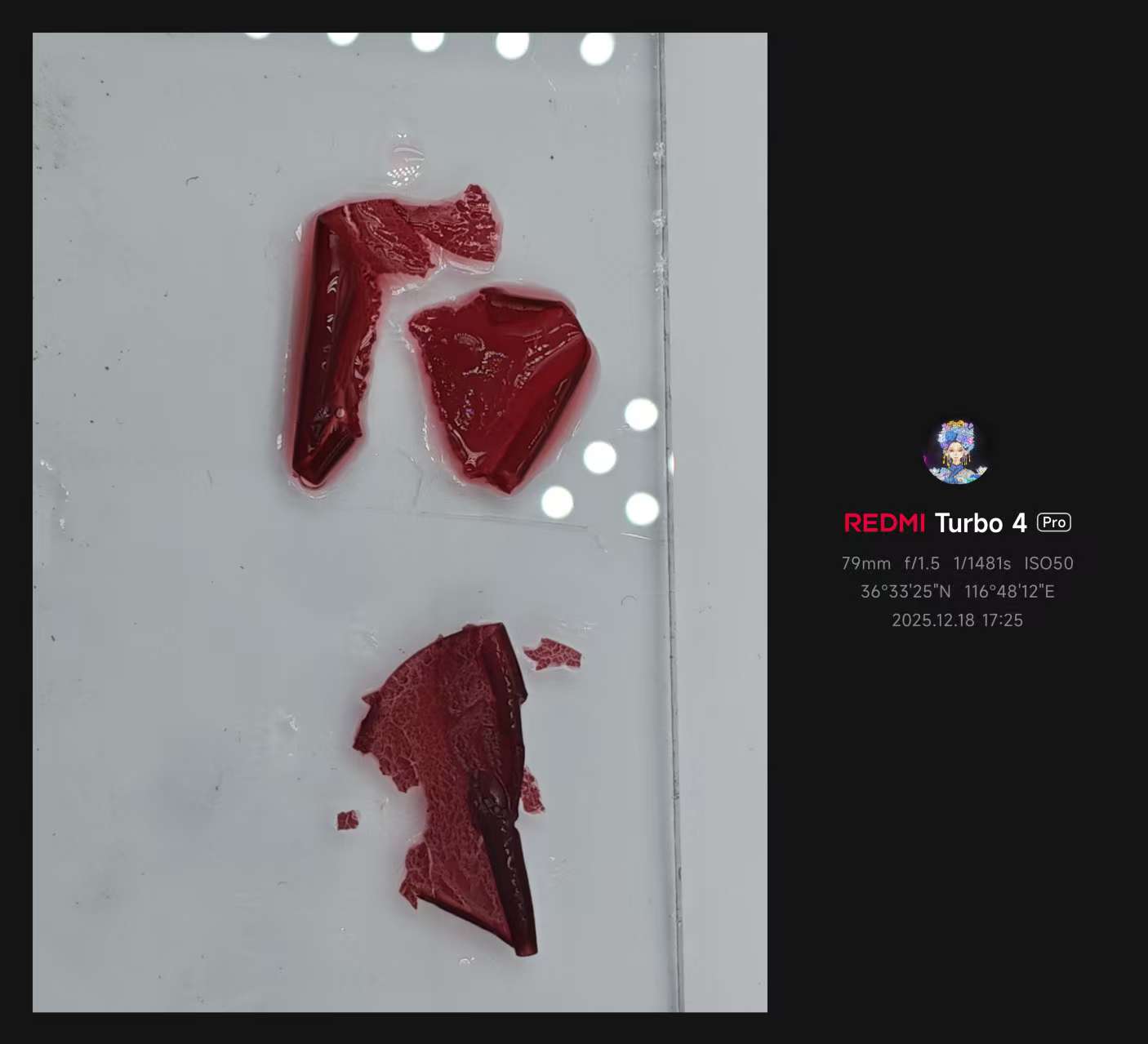 | 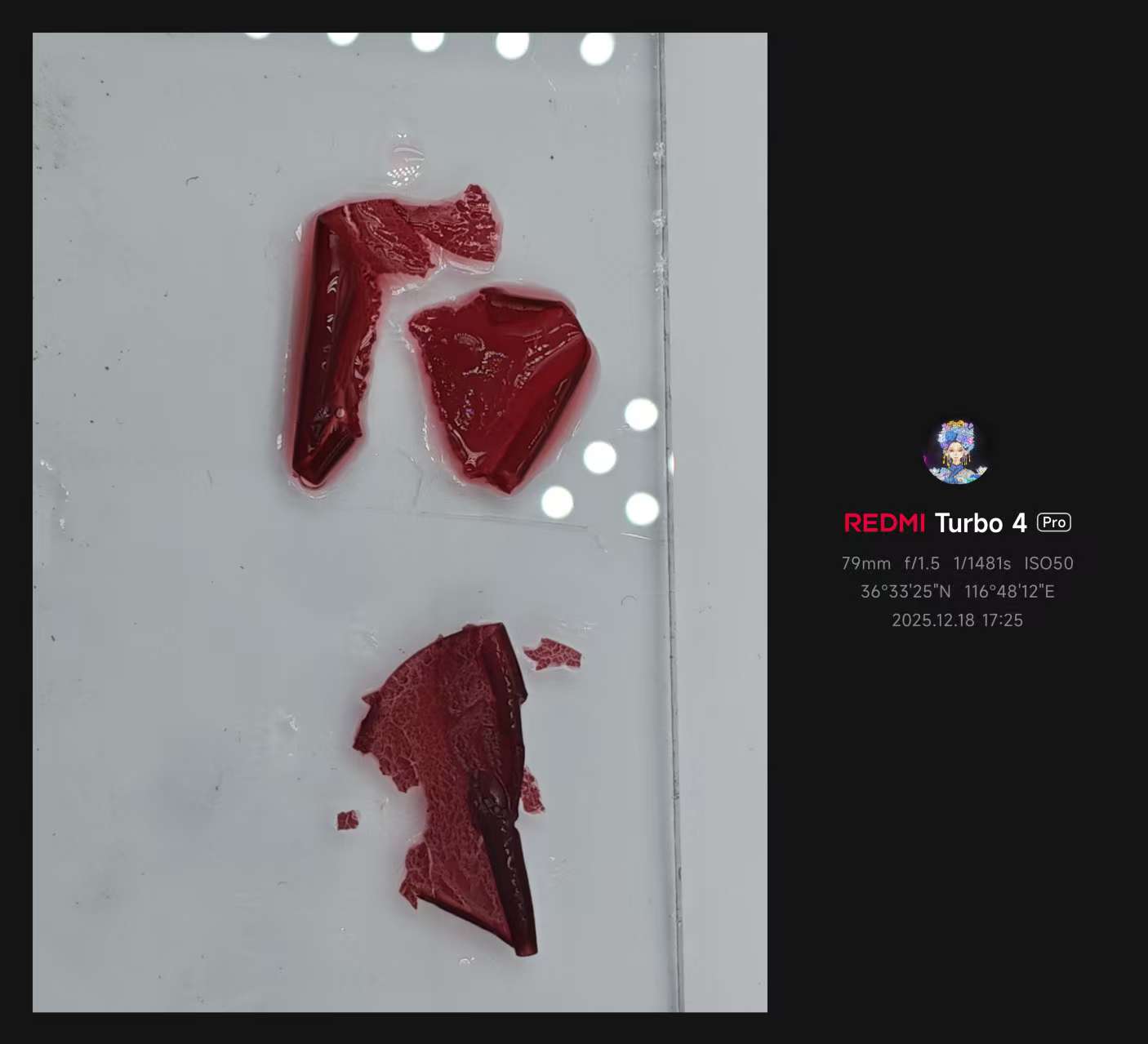 | 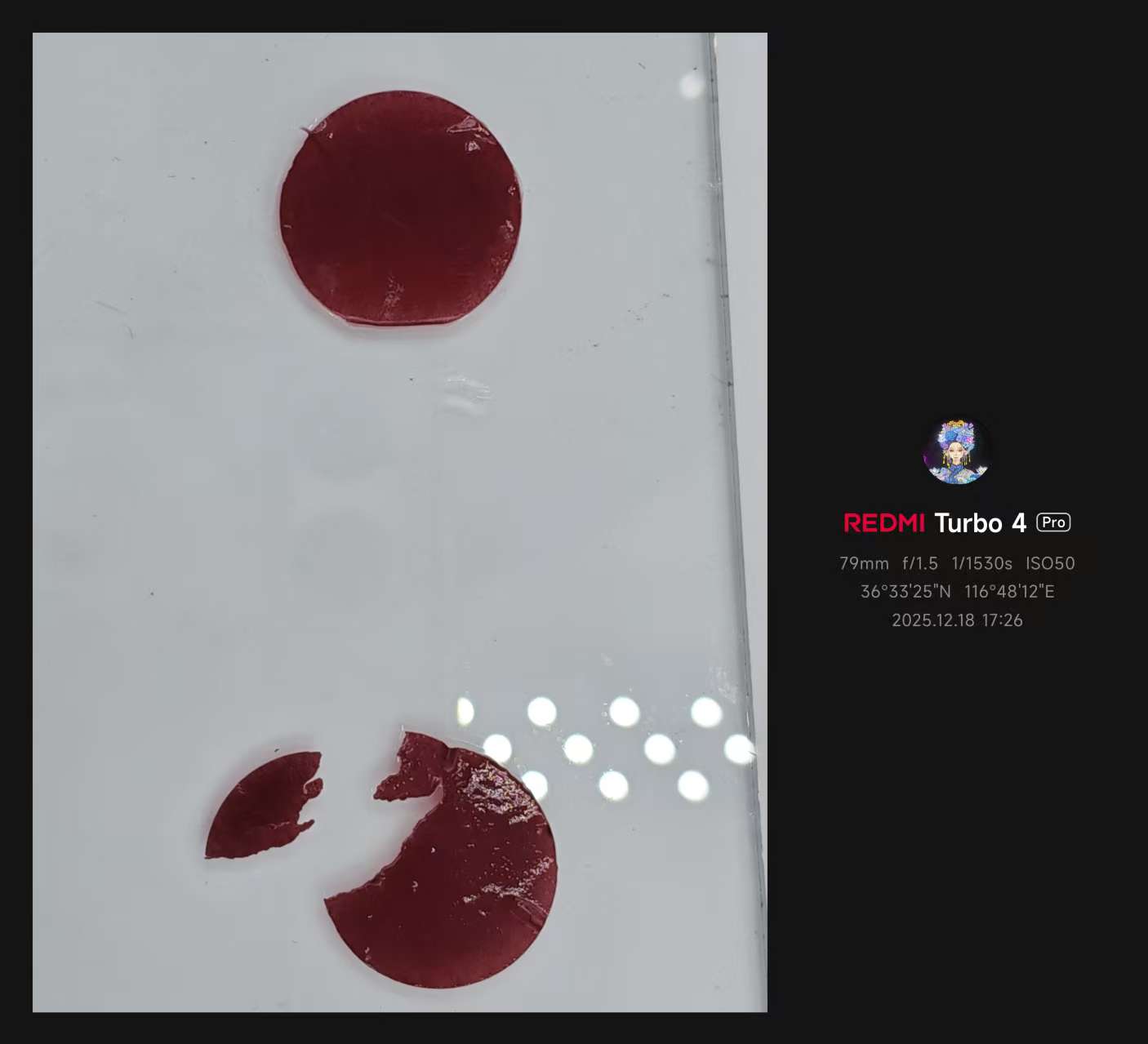 | 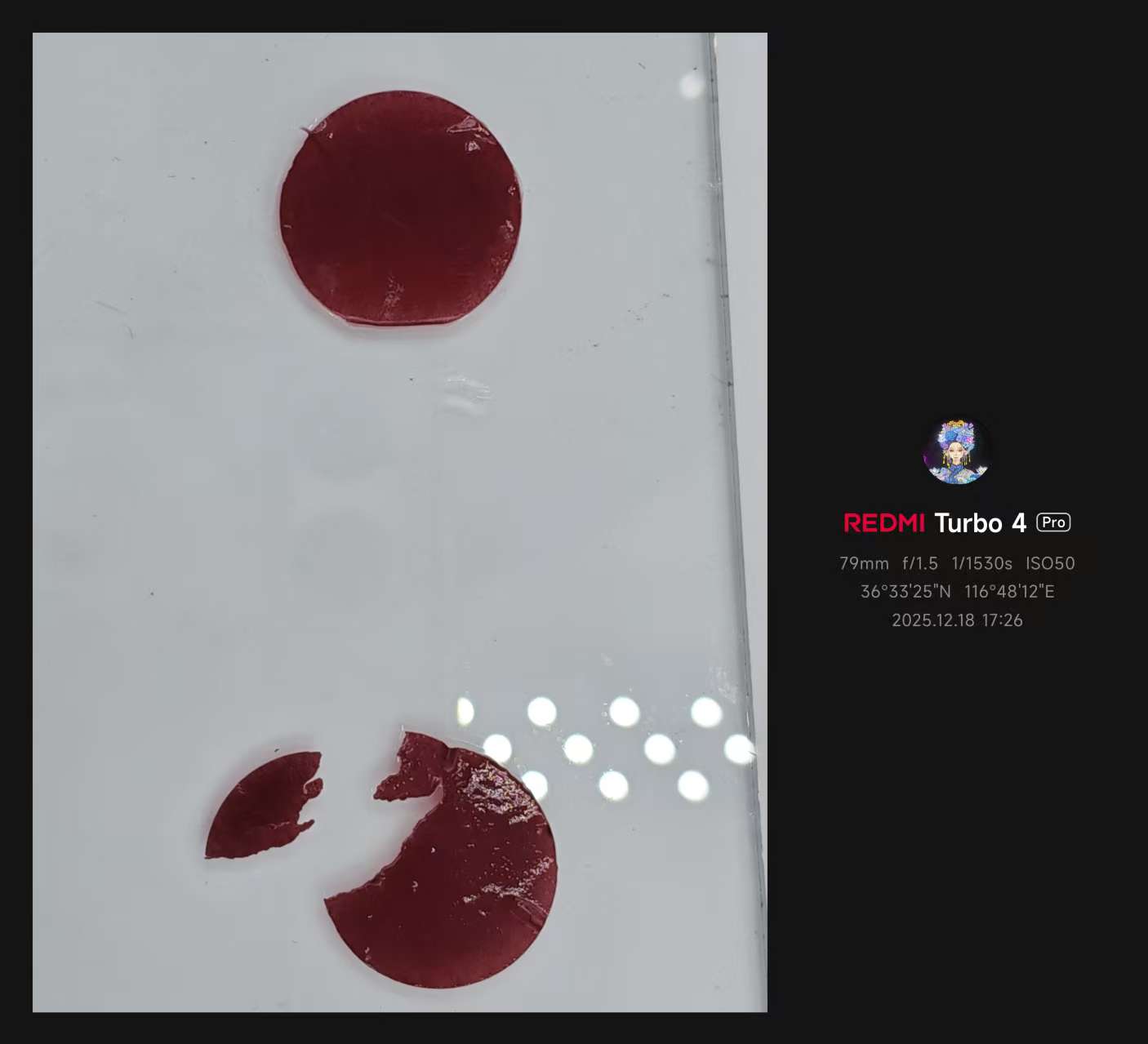 | 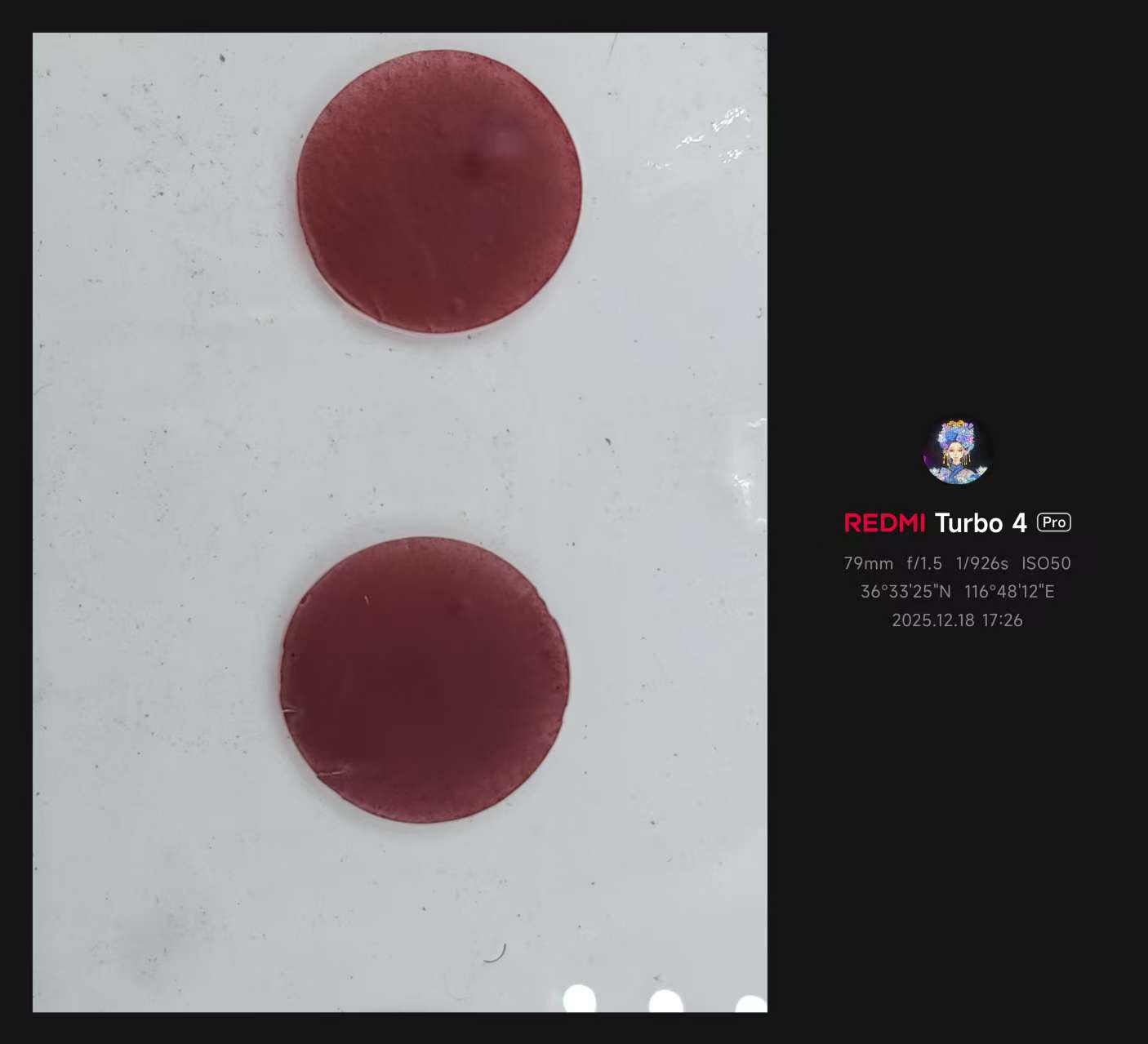 | 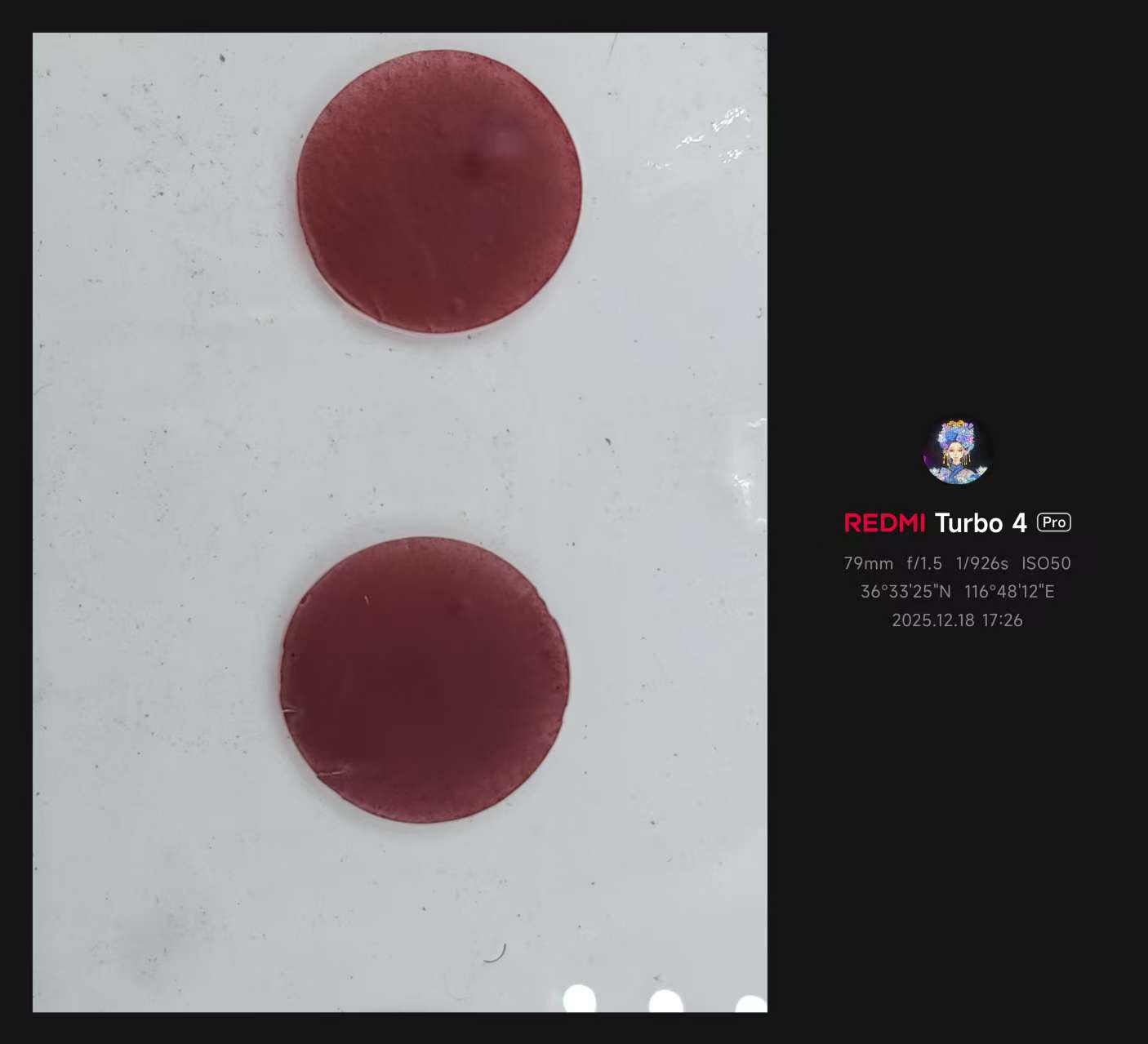 | 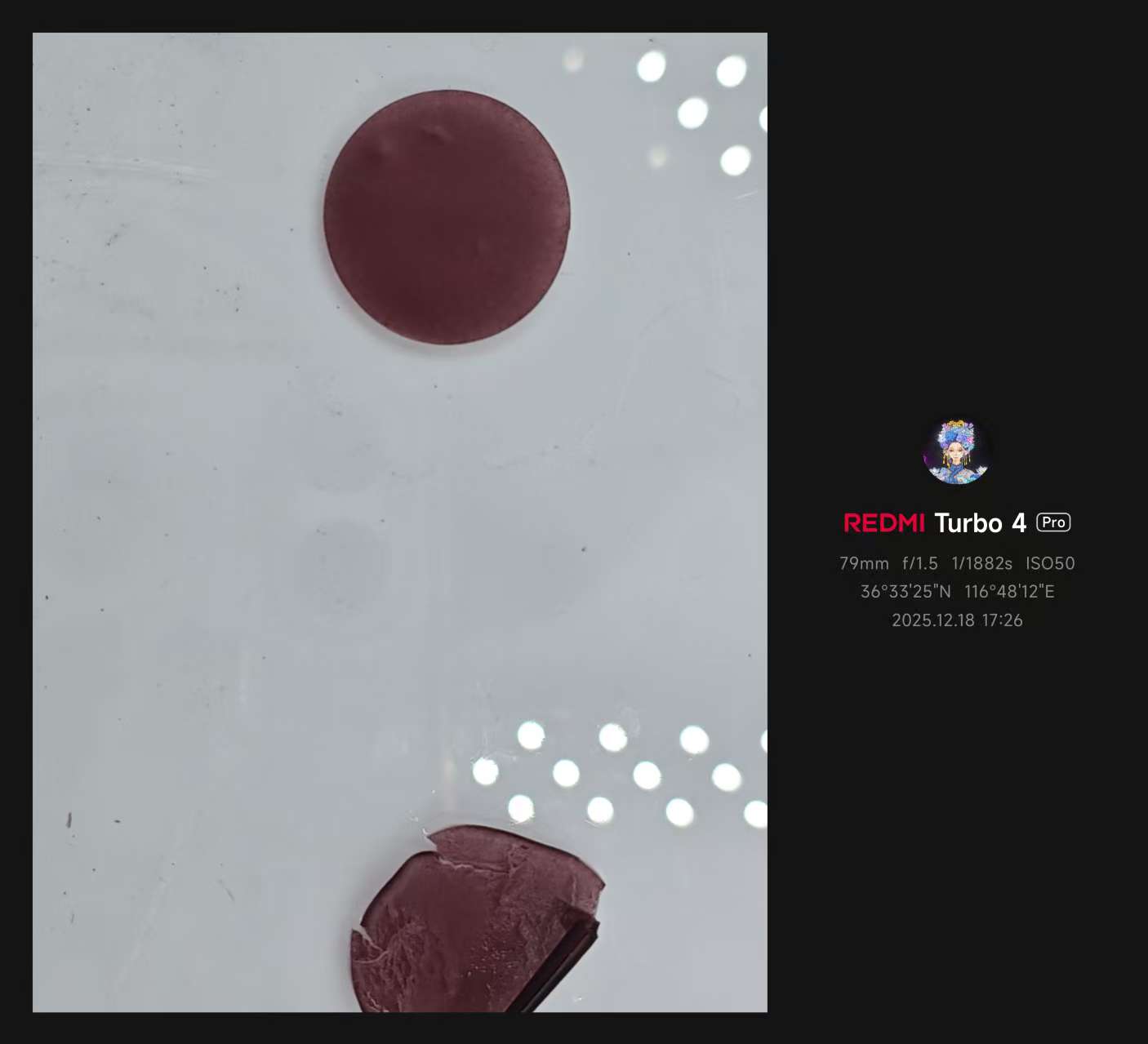 | 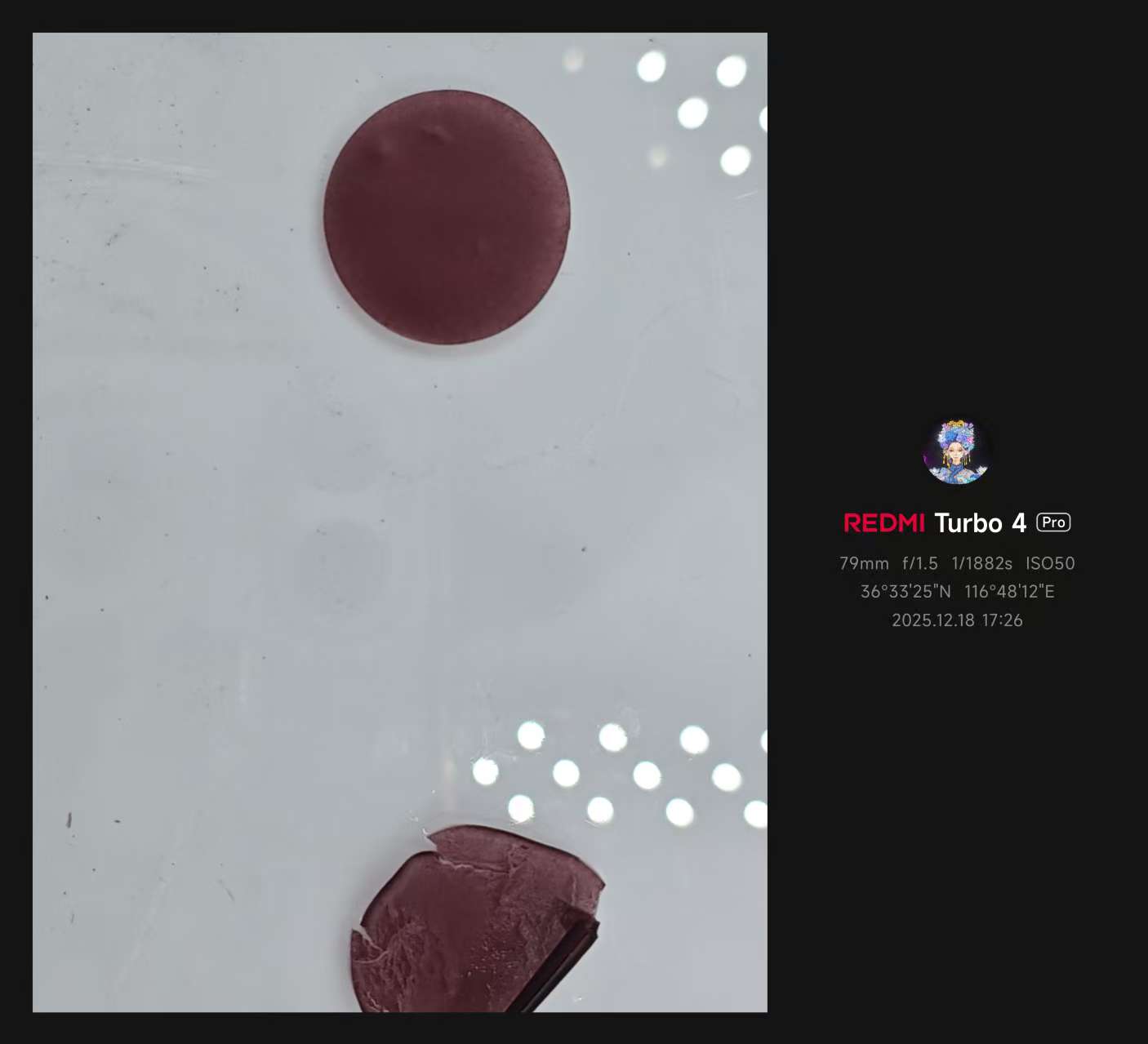 | 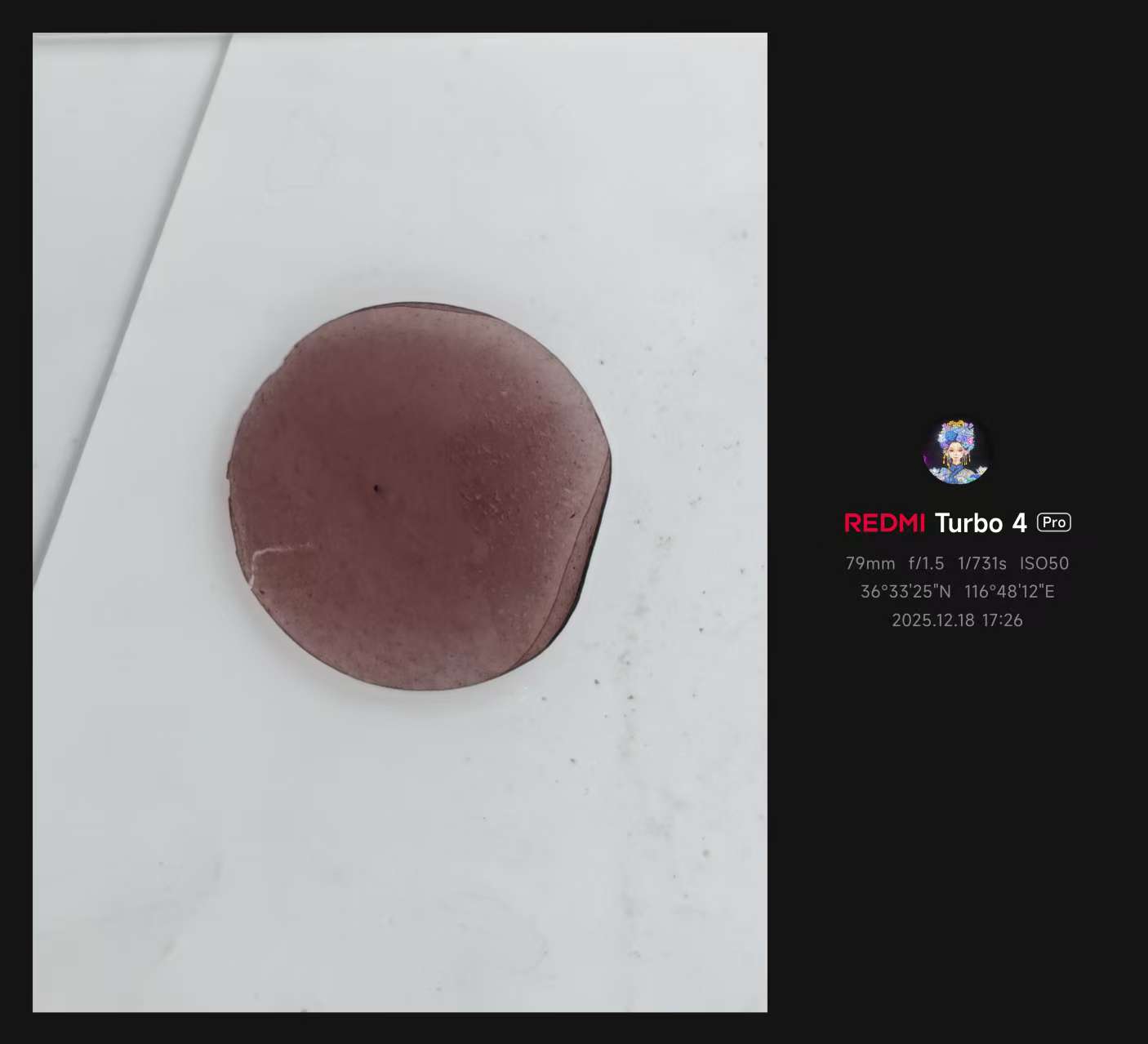 |
|  | L* | 17.1 | 14.9 | 14.6 | 15.3 | 16.3 | 14.4 | 16.9 | 16.5 | 22.3 |
|  | a* | 33 | 22.2 | 23.9 | 25.3 | 23 | 18.6 | 14.4 | 12.5 | 6 |
|  | b* | 12.4 | 5.2 | 7.5 | 8.5 | 6.9 | 5.9 | 3.9 | 4.3 | 4.5 |
|  | ΔE | 9.88 | 6.63 | 3.73 | 2.76 | 5.18 | 8.13 | 16.29 | 13.7 | 24.36 |
